# Coding relationship links RNA G-quadruplexes and protein RGG motifs in RNA-binding protein autoregulation

**DOI:** 10.1073/pnas.2413721122

**Published:** 2025-01-23

**Authors:** Marlene Adlhart, Daniel Hoffmann, Anton A. Polyansky, Bojan Žagrović

**Affiliations:** ^a^Max Perutz Labs, Vienna Biocenter Campus, Vienna 1030, Austria; ^b^Department of Structural and Computational Biology, University of Vienna, Vienna 1030, Austria

**Keywords:** RNA G-quadruplex, RGG motif, RNA-binding proteins, autoregulation, genetic code

## Abstract

RNA G-quadruplexes (rG4s), the four-stranded structures formed by guanine-rich RNA sequences, are recognized by regions in RNA-binding proteins (RBPs) that are enriched in arginine-glycine repeats (RGG motifs). Importantly, arginine and glycine are encoded by guanine-rich codons, suggesting that some RGG motifs may both be encoded by and interact with rG4s in autogenous messenger RNAs (mRNAs). By analyzing transcriptome-wide rG4 datasets, we show that hundreds of RGG motifs in humans are at least partly encoded by rG4s, with an increased incidence for longer RGG motifs (~10 or more residues). Using randomized genetic codes, we demonstrate that the rG4/RGG coding relationship derives from the universal genetic code’s structure. Moreover, we show that proteins, which contain RGG motifs encoded by experimentally detected rG4s, are significantly enriched in RNA binding relative to all RGG-containing proteins. Finally, using enhanced crosslinking and immunoprecipitation (eCLIP) data, we identify several prominent RBPs, including FUS, FMRP, and G3BP1, which interact with autogenous mRNAs in regions where RGG motifs are encoded by rG4s. Our results define a physically realistic mechanism behind autogenous mRNA/protein interactions that is hardwired in the genetic code structure and may contribute to the establishment of autoregulatory feedback loops in the cell.

## Significance

Many RNA-binding proteins (RBPs) undergo autoregulation by interacting with the messenger RNAs (mRNAs) that encode them, but such behavior is not well understood. Here, we uncover a potentially widespread mechanism behind autoregulatory mRNA–protein interactions, defined by the structure of the genetic code. Specifically, arginine-glycine repeats (RGG motifs) are well-known RNA-binding domains which interact with RNA G-quadruplexes (rG4s), compact structures formed by guanine-rich RNA sequences. We show that hundreds of RGG motifs in humans are encoded by rG4s in their own mRNAs and identify several RBPs where autogenous mRNA–protein binding occurs in regions linked by coding. Our results suggest that coupled coding and binding, as demonstrated for rG4/RGG motifs, may represent an important contribution to the autoregulatory behavior of RBPs.

RNA G-quadruplexes (rG4s) are compact, four-stranded RNA structures frequently adopted by guanine-rich sequences (1). Specifically, rG4s are formed by stacks of guanine quartets arranged in a planar geometry and stabilized by Hoogsteen base pairs and a central monovalent cation (1). These structures are thought to play important regulatory roles in various biological processes including transcription, splicing, translation, and messenger RNA (mRNA) degradation (1–3). Consequently, extensive efforts have been devoted to experimental identification and computational prediction of rG4s, providing evidence of their high prevalence in humans (1, 2, 4–6). Sequences with a high propensity toward rG4s have been detected in both coding and noncoding RNAs and are especially enriched in mRNA regulatory regions (2, 3, 7–9). Although their capacity to stably form in vitro has long been recognized, the existence of rG4s in vivo has been debated. Based on dimethyl sulfate (DMS) modification and reverse-transcriptase stalling assays, Guo and Bartel have suggested that rG4s tend to be largely destabilized in mammalian and yeast cells (8). This was attributed to cellular machinery that actively detects and unwinds rG4s (8). On the other hand, hundreds of folded rG4s could be detected in plant cells, with differences in temperature and K^+^-levels as compared to mammalian cells thought to contribute (10, 11). Importantly, rG4 folding is promoted in human cells under different stress conditions, impacting the stability of rG4-containing transcripts and potentially contributing to the formation of stress granules, P-bodies, and other biomolecular condensates (12, 13). Collectively, recent data paint a complex picture whereby rG4s undergo continuous unfolding and refolding, with their dynamics and functionality largely depending on the cellular context and interaction with rG4-binding proteins (13–18).

**Fig. 1.**
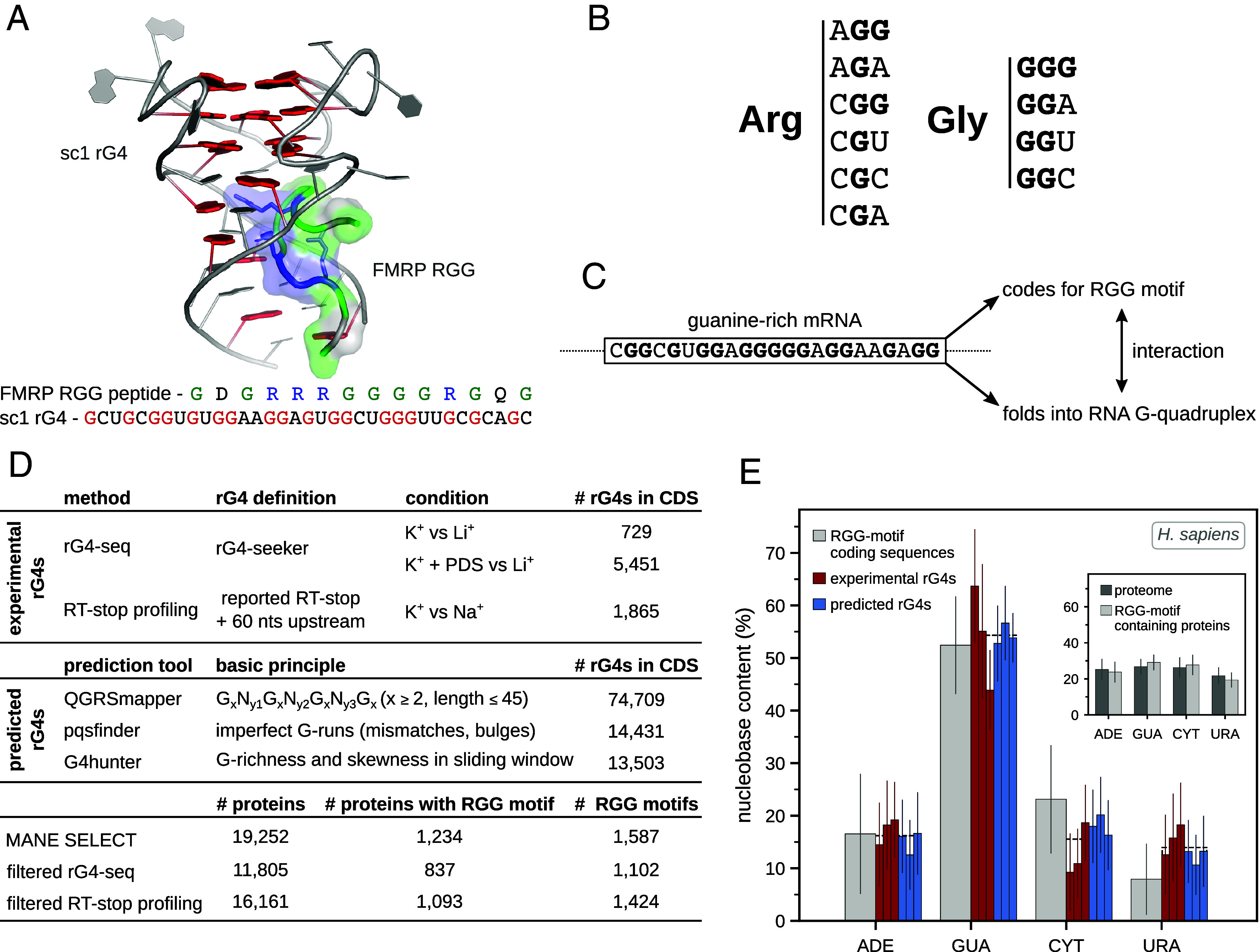
RGG motifs are encoded by guanine-rich sequences. (*A*) Interaction between FMRP RGG peptide and sc1 rG4 (PDB 5DE5) (38). Guanines are depicted in red, with arginines and glycines of the FMRP peptide given in blue and green, respectively. Sequences of the FMRP peptide and sc1 rG4 are given below the structure. (*B*) Codons coding for arginine and glycine. (*C*) A guanine-rich sequence may simultaneously have a high propensity to fold into an rG4 and encode an RGG motif, which in turn could bind to the rG4 that encodes it. (*D*) Summary of data used for analysis, including general information about experimental rG4 datasets and prediction tools used, number of nonoverlapping rG4s detected in MANE SELECT human transcript CDS regions, number of transcript/protein sequences, and number of detected RGG motifs. (*E*) Average nucleobase content of human RGG-motif coding sequences (gray) and nucleobase content of experimentally determined (red) and predicted (blue) rG4s [from *Left* to *Right*: rG4-seq (K^+^), rG4-seq (K^+^ + PDS), RT-stop profiling, QGRSmapper, pqsfinder, and G4hunter]. The average nucleobase content across the six different datasets is indicated with a dashed line. In the *Inset*, the average nucleobase content of complete coding sequences of all MANE SELECT proteins (dark gray) as well as complete coding sequences of RGG-motif-containing proteins (light gray) is given. Error bars indicate ±1 SD across genes.

Over the years, an increasing number of rG4-binding proteins have been detected via biochemical assays (18–20). In human alone, more than 100 of such proteins could be identified (3, 20), with several databases devoted to the topic (21, 22). Recently, a computational model to evaluate protein rG4-binding propensity has been developed based on experimental data (G4-FUNNIES) (20). Using the model, it was shown that rG4-binding proteins exhibit increased tendency toward structural disorder and biomolecular condensate formation (20). Importantly, by analyzing the amino acid composition of 77 RNA and DNA G-quadruplex binding proteins, Brázda et al. have shown that they are significantly enriched in arginine and glycine (23). In particular, the known arginine- and glycine-rich RNA binding motif (RGG motif) is frequently found in rG4-binding proteins (14, 18, 23). The RGG motif is typically characterized by several RG and/or RGG repeats, interspersed by short stretches of other amino acids. It is the second-most common RNA-binding motif known in humans, but it also mediates protein–protein interactions (24, 25). Being intrinsically disordered and highly flexible, the RGG motif adopts various conformations upon interaction with different nucleic-acid or protein targets (24–27). The essential role of the RGG motif for RNA and/or DNA G-quadruplex binding has been experimentally confirmed for multiple proteins, including fused-in-sarcoma (FUS) protein (28), Ewing sarcoma RNA binding protein 1 (EWSR1) (29, 30), cold inducible RNA binding protein (CIRBP) (31), nucleolin (NCL) (32, 33), G3BP stress granule assembly factor 1 (G3BP1) (34), heterogeneous nuclear ribonucleoprotein A1 (hnRNPA1) (35), methyltransferase 14 (METTL14) (36) and fragile X mental retardation protein (FMRP) (37–39). Moreover, the high-resolution NMR and crystal structures of the RGG peptide of FMRP bound to a synthetic rG4 structure designed by SELEX (sc1), have provided an atomistic picture of how such an interaction can be realized (38, 40). Specifically, it was shown that the FMRP RGG peptide adopts a type I β-turn fitting into the duplex-quadruplex junction of sc1, making contacts with the bases in both the duplex and the G-quadruplex of sc1 (Fig. 1*A*) (38, 40).

In addition to their binding relationship, rG4 and RGG motifs are potentially linked in another, less-well-characterized way. Namely, a cursory look at the genetic code reveals that arginines and glycines are heavily encoded by guanine-rich codons (Fig. 1*B*). Specifically, arginine is encoded by 6 and glycine by 4 codons with an average guanine content of 44% and 75%, respectively. Importantly, this implies that an RGG motif in a protein sequence may have an increased probability of being encoded by an rG4-forming mRNA region and vice versa. Combined with the fact that rG4 and RGG motifs are known interacting partners, this suggests a simple, genetically hardcoded mechanism behind a number of known autogenous interactions between mRNAs and the proteins they encode (Fig. 1*C*) (41). The main aim of the present study is to systematically evaluate this possibility. In general, autogenous interactions represent one of the simplest and the most ubiquitous regulatory paradigms in the cell (41–43). From viral (44, 45) and ribosomal proteins (46, 47) to transcription factors (48), from enzymes (49, 50) to splicing factors (51–53), the activity of many proteins is controlled by positive and negative autoregulatory feedback loops established by binding to their own mRNAs (41, 42). Moreover, a comprehensive analysis of crosslinking and immunoprecipitation with sequencing (CLIP-seq) data has shown that hundreds of RNA-binding proteins (RBPs) interact with their own autogenous mRNAs, with a significant preference for coding-sequence (CDS) binding (54). Importantly, such interactions have been suggested to potentially reflect the driving forces behind the origin of the universal genetic code (“coding from binding”) (55–58).

FMRP provides a paradigmatic example in this context. Specifically, one of the many RNA targets of this translational repressor is also its own autogenous mRNA (59, 60). Importantly, Blice-Baum and Mihailescu have shown that the major isoform of FMRP binds with a K_D_ of ~120 nM to an rG4-containing region in its own mRNA, which in turn encodes an RGG motif. This binding is even stronger for the minor FMRP isoforms ISO2 and ISO3 (K_D_ of ~65 nM) and provides a mechanistic foundation for the alternative splicing of FMRP pre-RNA and the ensuing feedback loop that controls FMRP isomer levels (61, 62). Furthermore, using NMR and gel-shift assays, Blice-Baum and Mihailescu have demonstrated that the FMRP RGG peptide interacts with a stable rG4 in the FMRP mRNA CDS located between positions 1602 and 1616, which directly encodes four glycines in the central part of the RGG box. To the best of our knowledge, this is the only example of an rG4 and an RGG motif related by coding, whose interaction has been characterized in significant detail. However, even in this case, the connection with the structure of the genetic code and its potential implications were not considered. Richard et al. (63) have commented on the potential significance of such a connection, but have used a search for rG4 sequences in human mRNA CDS regions to specifically analyze the *nonautogenous* mRNA binding partners of an RGG-containing cell-survival protein Aven, and in particular, mRNAs of mixed-lineage leukemia (MLL) proto-oncogenes 1 and 4.

Here, we use a transcriptomic-level analysis to characterize the potential for autogenous interactions between rG4 and RGG motifs in humans. Specifically, we comprehensively evaluate the overlap between regions in human protein-coding transcripts that form rG4s and those that encode RGG motifs (Fig. 1*D*) and link it with the structure of the genetic code. We identify hundreds of RGG motifs that are partly encoded by rG4s and cross-reference our results with the extant enhanced crosslinking and immunoprecipitation (eCLIP) data. As its principal contribution, our study defines a mechanistic principle embedded in the structure of the genetic code, which could underlie the interaction between many RBPs and their autogenous mRNAs and thus contribute to the formation of cellular autoregulatory circuits.

## Methods

### Transcript Data Selection.

MANE SELECT (v1.2) (64) was used to define and select the dominant human transcripts, corresponding to a total of 19,252 protein-coding genes that were analyzed. For the analysis of experimental rG4 data, this set was filtered to remove genes that were likely not expressed in the corresponding experiments (for details, see *Filtering of Transcript Data for Experimental rG4s*).

### Analysis of Experimentally Determined rG4s.

Three different experimental in vitro rG4 datasets, all of which exploited reverse-transcriptase stalling (RTS) to detect rG4s, were used for the analysis: 1) rG4-seq data (GSE77282), obtained by Kwok et al. using HeLa cells (7) in the presence of K^+^, in combination with a subsequently introduced *rG4-seeker* analysis pipeline (65), 2) the same as 1, but in the presence of K^+^ + pyridostatin (PDS, an rG4 stabilizing agent), and 3) RT-stop profiling data (GSE83617) obtained using HeLa and HEK293T cells by Guo et al. (8). While each of these strategies compares RTS under rG4 stabilizing (K^+^) and less stabilizing conditions (Li^+^, Na^+^) and identify rG4s as those RTS sites that are detected under rG4 stabilizing conditions only, they exhibit relevant differences in both experimental procedures and computational analysis used (reviewed in ref. 4). Importantly, Kwok et al. identified and reported consensus rG4s that are detected in at least 2 out of 4 studied samples and under *both* K^+^ and K^+^ + PDS conditions (65). However, we reasoned that rG4s that can only be detected under strong rG4 stabilizing conditions (K^+^ + PDS) could be of relevance as well, given the dynamic nature of rG4s and the known dependence on the stabilizing interactions with proteins or other factors in the cell. As a case in point, the biophysically and functionally well-characterized rG4 in the CDS of FMRP mRNA (59, 61, 62) did not pass the original criteria and was not reported by Kwok et al. (65). For this reason, we have reanalyzed their data using *rG4-seeker* (65) following the same approach, but relaxing the criteria and reporting rG4s if they are detected in at least 2 out of 4 samples under either K^+^ or K^+^ + PDS conditions, or both. Specifically, the sequencing reads were quality- and adapter-trimmed using trim-galore (66) and aligned to the human reference genome (GRCh38) using STAR aligner (67). Uniquely aligned reads were then used for analysis with the publicly available *rG4-seeker* software (65), with GENCODE v.45 (68) annotations for definition of transcriptomic regions. For subsequent analysis, only rG4s that mapped to MANE SELECT (64) transcripts were retained.

For the analysis of RT-stop profiling data, for which only one sample was studied per cell line, we have combined data from HeLa and HEK293T cell lines, used RT-stop positions as reported by the authors, mapped them to the MANE transcript sequences and considered the region 60 nucleotides upstream as an rG4 region, as defined by the original authors (8).

### Filtering of Transcript Data for Experimental rG4s.

In order to filter the transcripts for those that were at all available in the experiment and use them as a background in subsequent analysis, the sequencing reads of the rG4-seq data, which were used for the analysis with rG4-seeker, were quantified using *featureCounts* (69) and genes with a CPM ≥ 1 in at least two samples and under Li^+^ and K^+^ or K^+^ + PDS conditions were retained. For RT-stop profiling, raw sequencing reads were quantified using *kallisto* (v0.50.1) (70) and transcript abundances (in TPM) were summed to obtain gene abundances. The genes that had a TPM ≥ 0.5 in both stabilizing (K^+^) and less-stabilizing (Na^+^) conditions in the single sample of at least one cell line (HEK293T or HeLa) were retained. In this way, the number of transcripts used for the analysis was reduced from 19,252 to 12,138 for the two rG4-seq datasets and 16,161 for the RT-stop profiling dataset.

### Computational Prediction of rG4s.

Three different prediction tools based on different approaches (Fig. 1*D*) were used in order to computationally predict rG4 locations: QGRSmapper (71), pqsfinder (72), and G4hunter (73). Predictions were performed using coding sequences extended by the adjoining 50 nucleotides in 5′ and 3′ untranslated regions (UTRs), whenever possible. Following the approach adopted in an rG4 database QUADRatlas (22), the applied score thresholds used were 19, 47, and 1.2 for QGRSmapper, pqsfinder, and G4hunter, respectively, with default parameters otherwise.

### Definition of RGG Motifs.

Thandapani et. al have defined di- or tri- RGG motifs as protein sequences with either 2 or 3 consecutive RGG repeats, interspersed by 0 to 4 arbitrary amino acids, and have used an analogous definition for di- or tri- RG motifs (24). Combining these definitions, we consider protein sequences that have at least two consecutive RG *or* RGG repeats, interspersed by a maximum of 4 arbitrary amino acids, as RGG motifs (RG_1-2_[X_0-4_RG_1-2_]_1+_, where X stands for any amino acid). In this way, a total of 1,587 motifs are identified across 1,234 distinct human protein sequences, ranging between 4 and 52 amino acids in length (see Fig. 1*D* for further details). While the above definition also includes RG-only containing repeats, we refer to these as RGG motifs as well, following the accepted convention in the field.

### Overlap between rG4 and RGG Motifs.

An RGG motif is said to be partly encoded by an rG4 if the coding sequence of the protein motif has an overlap of 6 or more consecutive nucleotides with an rG4. Likewise, an rG4 is said to be partly coding for an RGG motif, if 6 or more consecutive nucleotides of the rG4 coincide with an RGG-motif coding sequence.

### Randomization of the Genetic Code.

The genetic code was randomized to either generate alternative translations while keeping the coding sequences (and rG4s) fixed, or to recode mRNA sequences while keeping the protein sequences (and RGG motifs) fixed. For the generation of alternative translations, the genetic code was randomized in two different ways. Specifically, either codons or amino acids in the genetic code table were randomly reassigned, while keeping the general structure of the code intact in both cases. In the first instance, the 61 protein-coding codons were randomly reassigned to amino acids such that the number of codons for each amino acid remained fixed and identical to the native genetic code, for a total of 10,000 times. In the second instance, codon boxes were assigned to different amino acids. As only the assignment of arginine and glycine are relevant for the determination of RGG motifs, all possible combinations of different assignments of these two amino acids were considered (379 alternative combinations + native genetic code). The original coding sequences were subsequently translated using the randomized genetic codes, and RGG motifs identified in the same way as for the native sequences. For each randomized code, the percentage of RGG motifs that are encoded by rG4s, as defined above, was determined.

For the generation of recoded mRNA sequences, the genetic code was randomized 1,000 times by shuffling the codons, following the same procedure as for generating randomized translations, as described above. In this way, each codon is unambiguously mapped to another codon. Coding sequences were subsequently rewritten using this mapping, while keeping the short UTR extensions fixed. rG4s were predicted for the recoded mRNAs in the same way as for the native sequences and the percentage of RGG motifs that are encoded by the predicted rG4s was determined for each randomized genetic code.

For all randomized backgrounds, the *P*-values associated with the percentage of RGG motifs encoded by rG4s were evaluated as the fraction of randomized genetic codes for which a greater or equal percentage of motifs encoded by rG4s was achieved as compared to the native genetic code.

## Results

### Nucleotide Content of RGG-Motif Coding Sequences Matches That of rG4s.

The notion that RGG-motif coding sequences and rG4s may be compositionally related was based on a qualitative observation that arginine and glycine codons are heavily enriched in guanine (Fig. 1*B*). This statement is surprisingly well supported by a quantitative analysis using natural sequences. When it comes to rG4s in coding sequences, their total number depends significantly on the method of detection and ranges anywhere between several hundred nonoverlapping rG4s for the rG4-seq (K^+^) dataset and tens of thousands for the QGRSmapper dataset (Fig. 1*D*). For this reason, we always present the results for individual methods separately, if not indicated otherwise. On the other hand, there exist 1,587 RGG motifs in the MANE SELECT human transcripts, with this number changing to 1,102 and 1,424 for the filtered rG4-seq and RT-stop profiling datasets, respectively (Fig. 1*D*). Importantly, the nucleotide composition of RGG-motif coding sequences closely matches that of rG4 regions, regardless of how rG4s are assigned (Fig. 1*E*). Specifically, the average guanine content in both RGG-motif coding sequences and rG4s hovers around 50%, with the other three nucleotides splitting the remainder approximately equally. The biggest deviation is seen for uracil, but even there it does not exceed ~6% in absolute units on average. While this matching does not imply that RGG motifs are significantly encoded by rG4s, it provides a quantitative foundation for this possibility, which we explore next.

### Hundreds of RGG-Motif Coding Regions Overlap with rG4s.

**Fig. 2.**
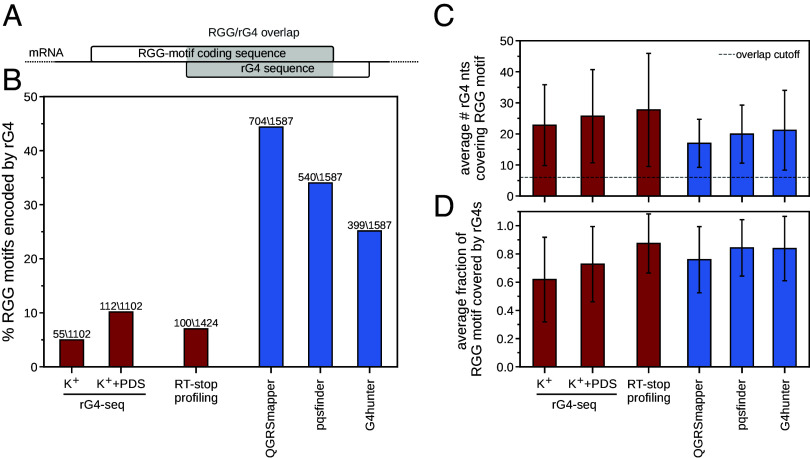
RGG-motif coding sequences overlap extensively with rG4s. (*A*) Overlap between RGG-motif coding sequences and rG4s was determined for the human transcriptome. An RGG motif is considered to be partly encoded by an rG4 if its coding sequence overlaps with an rG4 sequence over at least 6 consecutive nucleotides. (*B*) Percentage of RGG motifs in the human transcriptome that are partly encoded by rG4s as detected in the different experimental datasets (red) or predicted computationally (blue). The absolute numbers of RGG motifs partly encoded by rG4s, as well as the total number of RGG motifs in the corresponding background datasets are shown on *Top* of the bars. (*C*) Average number of rG4 nucleotides that overlap with an RGG-motif coding sequence and (*D*) average fraction of RGG-motif length that is covered by rG4 nucleotides, for those RGG motifs that are partly encoded by rG4s. Error bars indicate ±1 SD.

We have assessed the incidence of RGG motifs that are partly encoded by rG4s in the human protein-coding transcriptome for different rG4 datasets (Fig. 2*A*; see *Methods*). Overall, we identify 115 RGG motifs that are partly encoded by rG4s as detected by rG4-seq, corresponding to approximately 10% of all RGG motifs in our analysis. Among those, 55 are identified under K^+^-only conditions and 112 under K^+^+PDS conditions, with the latter largely extending the set of rG4s that can be detected under K^+^-only conditions (Fig. 2*B* and *SI Appendix*, Fig. S1). Similarly, 100 RGG motifs show overlap with rG4 regions identified by RT-stop profiling (7%). In general, a reasonable agreement between the different experimental datasets is observed, with 57 RGG motifs partly encoded by rG4s detected in both the pooled rG4-seq dataset and the RT-stop profiling dataset, and 158 RGG motifs partly encoded by an rG4 in at least one of the two datasets (*SI Appendix*, Fig. S1).

When it comes to the computationally predicted rG4s, a total of 399 (G4hunter), 540 (pqsfinder), and 704 (QGRSmapper) RGG motifs are encoded in part by an rG4, corresponding to 25%, 34%, and 44% of all RGG motifs, respectively (Fig. 2*B*). Overall, among all RGG motifs, 901 (57%) are partly encoded by an rG4 that was predicted by at least one tool, while 226 (14%) are encoded by rG4s that were predicted by all 3 tools (*SI Appendix*, Fig. S1). Regarding the agreement between experimentally and computationally determined rG4s, anywhere between 47% and 76% of the 158 RGG motifs that are partly encoded by experimentally determined rG4s also show overlap with a predicted rG4, depending on the tool used. Having said this, 91% of those overlap with an rG4 predicted via at least one tool (*SI Appendix*, Fig. S1).

The minimal overlap of six consecutive nucleotides guarantees that at least a single RG or GG dipeptide is encoded by a fragment of an rG4, but one could argue that this criterion is relatively lenient. Importantly, we observe in practice a significantly greater intersection between RGG-motif coding sequences and rG4s as compared to this minimal requirement, with an average overlap in different datasets ranging from ~17 to 28 nucleotides (Fig. 2*C*). In other words, the RGG motifs that are labeled as being partly encoded by rG4s are on average significantly encoded by rG4s, with the average coverage of RGG motifs by rG4 sequences ranging from 62 to 87% (Fig. 2*D*). Finally, there is only a minor change in the fraction of rG4-encoded RGG motifs with an increase in the overlap cutoff e.g., a decrease of 4 to 9% for predicted and 0.6 to 1.5% for experimentally identified rG4s, when increasing the cutoff from 6 to 12 nucleotides, the minimum length of an RGG-motif coding sequence in our analysis (*SI Appendix*, Fig. S2*A*). However, the agreement between different experimental and computational rG4 datasets remains largely unchanged (*SI Appendix*, Fig. S2*B*).

In general, the difference in the number and the identity of RGG motifs characterized as being partly encoded by rG4s detected via different methods can be attributed to the fact that they differ significantly in their identification of rG4s (Fig. 1*D*). Interestingly however, while the overlap between nucleotides belonging to rG4s across all coding sequences is limited to 5 to 6% for the experimental and computational datasets analyzed, this number increases ~fourfold when considering rG4 sequences within RGG-motif coding sequences only (*SI Appendix*, Fig. S3*A*).

### Long and Compact RGG Motifs Are Frequently Encoded by rG4s.

We have next analyzed the specific features of the RGG motifs that are encoded by rG4s. Across all experimental and computational rG4 datasets analyzed, these motifs tend to be longer as compared to those for which no such connection is detected (*SI Appendix*, Fig. S4 *A* and *E*). As the number of RG and/or RGG repeats scales with motif length (*SI Appendix*, Fig. S4*C*), we find that the number of RG as well as RGG repeats in a given motif is significantly higher for motifs partly encoded by rG4s (*SI Appendix*, Fig. S4 *B* and *E*). Additionally, linker sequences between the repeats tend to be shorter in those motifs, indicating that especially compact motifs with a high number of densely packed RG/RGG repeats tend to be encoded by rG4s (*SI Appendix*, Fig. S4 *B* and *E*). This is further illustrated for RGG motifs with a fixed length, for which a significantly higher number of RGG repeats as well as shorter linker sequences are found for rG4-encoded motifs for most methods (*SI Appendix*, Fig. S4 *D* and *F*). Additionally, consistent differences between the amino acid composition of linker sequences in rG4-encoded RGG motifs and those not encoded by rG4s indicate that particularly glycine-rich linkers contribute to the observed coding relationship (*SI Appendix*, Fig. S4 *G* and *H*). Notably, while this trend cannot be observed using predicted rG4s, the linker sequences within RGG motifs encoded by experimentally detected rG4s are also rich in phenylalanine and tyrosine, amino acids in RGG motifs shown to play important roles for G-quadruplex binding (31, 33, 74) (*SI Appendix*, Fig. S4 *G* and *H*).

**Fig. 3.**
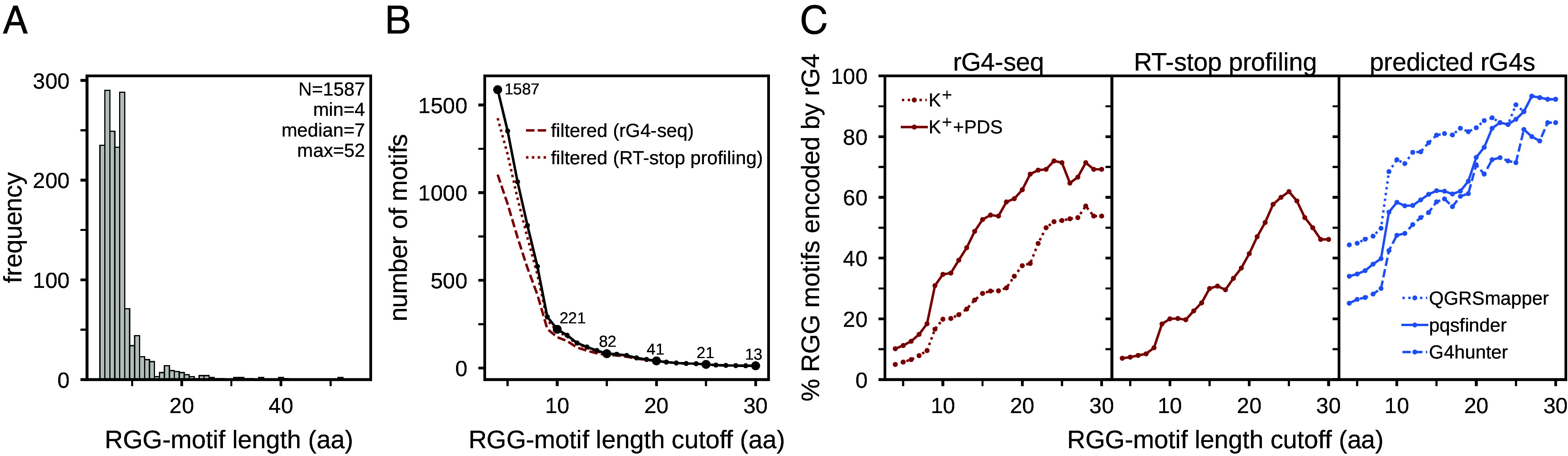
Longer RGG motifs are frequently encoded by rG4s. (*A*) Distribution of RGG-motif lengths among MANE SELECT proteins. (*B*) Number of RGG motifs in the complete dataset and the two expression-filtered datasets as a function of motif-length cutoff. (*C*) Percentage of RGG motifs partly encoded by rG4s as a function of motif-length cutoff.

Owing to the adopted definition of such motifs from Thandapani et al. (24) and a higher abundance of shorter motifs in the human proteome (Fig. 3*A*), the majority of RGG motifs included in our analysis are relatively short, with a median length of 7 amino acids. While it is unclear if these motifs can act as functional rG4-interacting motifs, proteins that have been confirmed to bind rG4s via their RGG-domains typically involve longer RGG motifs. For example, Brázda et al. have identified in rG4-binding proteins a shared sequence motif of length 20, containing multiple, closely spaced RG/RGG repeats with a glycine-rich linker sequence (23). Importantly, we identify similar characteristics in RGG motifs encoded by rG4-sequences. In fact, when only considering RGG motifs that are longer than or equal to a specific threshold (Fig. 3*B*), the percentage of rG4-encoded RGG motifs increases greatly after the cutoff of ~10 residues and continues to increase with motif length for all analyzed rG4 datasets, reaching ~50 to 70% for the experimentally determined and ~80 to 90% for the predicted rG4s at the motif length of 30 (Fig. 3*C*).

Remarkably, 40 out of the 41 RGG motifs with length ≥ 20 residues are partly encoded by an rG4 detected via at least one experimental or computational method, with the vast majority of them being encoded by rG4s identified in multiple datasets (*SI Appendix*, Fig. S5). As expected, the agreement concerning the RGG motifs that are encoded by rG4s as detected by different methods improves with motif length: longer RGG motifs tend to be encoded by rG4s that are detected by multiple methods more often (*SI Appendix*, Fig. S5). Moreover, the agreement between the experimentally detected rG4s within RGG-motif coding sequences improves for longer RGG motifs (*SI Appendix*, Fig. S3*B*).

### Proteins with RGG Motifs Encoded by Experimental rG4s Are Enriched in RNA Binding.

**Fig. 4.**
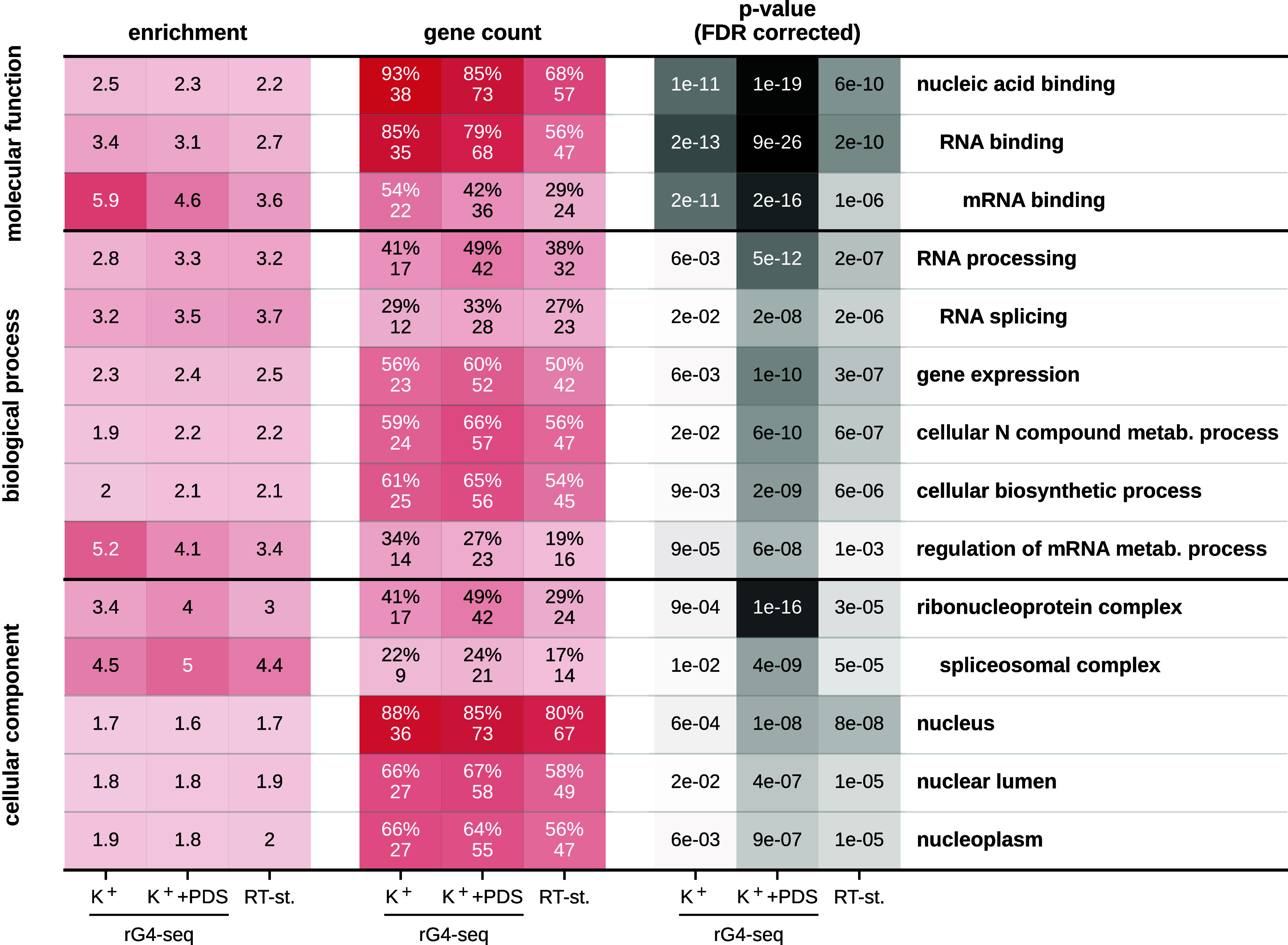
Proteins that contain RGG motifs encoded by experimentally determined rG4s are enriched in RNA binding. Significantly enriched GO-terms among proteins with RGG motifs that are partly encoded by experimentally determined rG4s, with the set of all RGG-motif-containing proteins in the expression-filtered datasets as the background. GO-enrichment analysis was performed using the PANTHER (v18.0) online tool (75), using Fisher’s exact test with FDR correction using the complete GO annotation datasets for the 3 GO categories, independently. The presented terms were selected as those with a corrected *P*-value of <0.05 for all three experimental datasets and a corrected *P*-value of <0.0001 for at least two of those datasets and manual curation to remove general high-level terms in order to decrease redundancy.

We have next performed Gene Ontology (GO) functional enrichment analysis using PANTHER v18.0 (75) in order to assess which, if any, biological functions are associated with proteins that contain rG4-encoded RGG motifs. Specifically, the overrepresentation analysis was performed using Fisher’s Exact test with false discovery rate (FDR) correction using complete GO annotation datasets for the three canonical GO representations independently. For each of the experimental and computational rG4 datasets, the proteins that contain RGG motifs encoded by rG4s were tested against all RGG-motif-containing proteins in the same dataset as the background. Importantly, one observes a strong pattern for proteins harboring RGG motifs encoded by experimentally identified rG4s (Fig. 4). In particular, for all experimental datasets, *RNA binding* ranks as one of the most significantly enriched GO terms for these proteins, as compared to all RGG-motif-containing proteins in the dataset as a background. The strongest enrichment of RNA binding was observed for rG4-seq (K^+^) data, with 35 out of the 41 proteins (85%) containing an rG4-encoded RGG motif annotated with the term *RNA binding* (Fig. 4). Moreover, some of the highest enrichments are observed for mRNA-binding and regulation of mRNA metabolic processes (Fig. 4), linking our findings to autoregulatory processes. Notably, none of the sets of proteins derived using the computational rG4 datasets are associated with significantly enriched GO terms.

### rG4/RGG Overlap Reflects Structure of the Genetic Code.

**Fig. 5.**
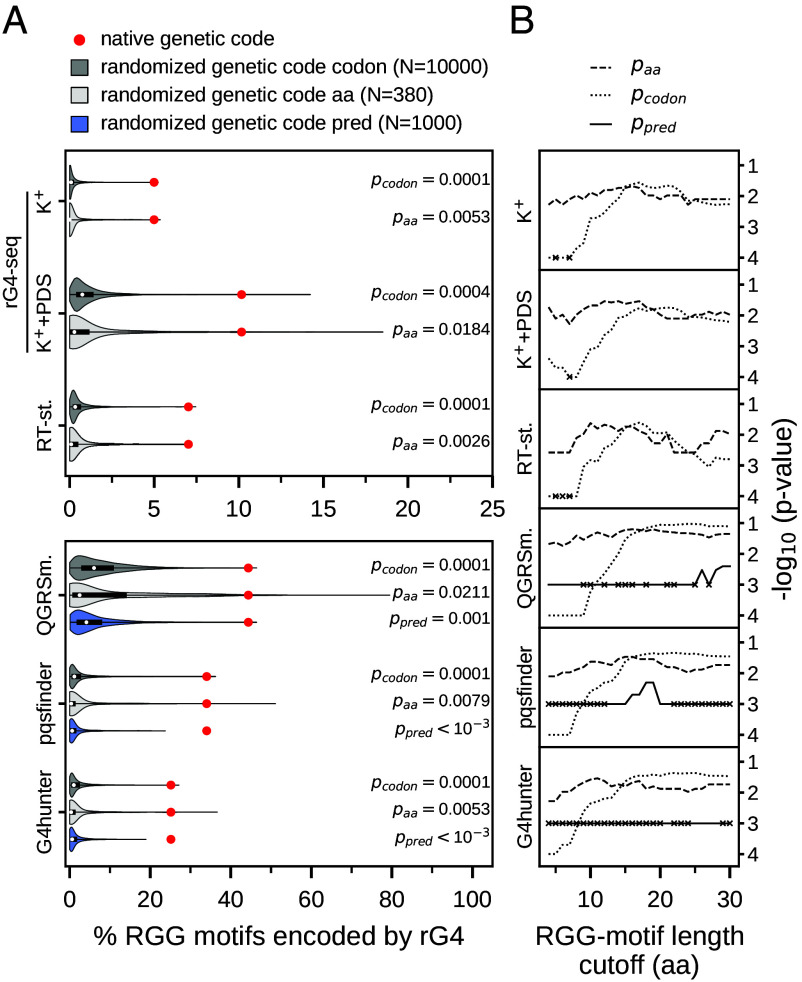
Overlap between RGG-motif coding sequences and rG4s is significant compared to alternative genetic codes. (*A*) Distribution of the proteome-wide fraction of RGG motifs that are partly encoded by rG4s, for different backgrounds obtained by using randomized genetic codes, for all rG4 identification methods. The value obtained for the native genetic code is depicted as a red dot. *P*-values associated with the corresponding randomized backgrounds are given on the *Right*. (*B*) Dependence of *P*-values for different backgrounds and rG4 identification methods on motif-length cutoff, with crosses indicating that none of the randomized genetic codes obtained a higher fraction of RGG motifs encoded by rG4s.

To evaluate the role of the genetic code structure in this relationship, we have next compared the number of RGG motifs that are partly encoded by rG4 sequences against different backgrounds. Specifically, we have randomized the genetic code in two different ways (*see Methods*) and translated all native coding sequences with these randomized genetic codes to obtain alternative proteomes. Within these protein sequences, we have searched for RGG motifs and, in each instance, determined the percentage of RGG motifs that are encoded by rG4s. Across the board, randomized genetic codes result in significantly fewer RGG motifs that are encoded by rG4s as compared to the native genetic code (Fig. 5*A*). For example, in the case of rG4s that were experimentally determined via rG4-seq (K^+^), only 1 out of 10,000 randomized codes obtained by codon shuffling led to a greater or equal overlap (p_codon_ = 0.0001) and 2 out of 380 codes generated by shuffling amino acids led to a greater or equal overlap than for the native code (p_aa_ = 0.0053) (Fig. 5*A*). Similar values are seen for both randomized backgrounds and all experimental and computational rG4 assignments used (Fig. 5*A*). Furthermore, for backgrounds where amino acids were assigned to native codon boxes at random, alternative codes that exhibit a percentage that is close to or greater than for the native code are almost exclusively the codes in which glycine codons are the same as the native ones for all experimental and prediction methods used (*SI Appendix*, Fig. S6).

In the case of predicted rG4s, we have additionally used randomized genetic codes to recode the native coding sequences, while keeping the protein sequences and RGG motifs fixed, and subsequently predicted rG4s in the recoded sequences. In agreement with the results obtained for randomized translations, the percentage of RGG motifs that are encoded by rG4s in the case of the universal genetic code is significantly greater than for randomized codes. Specifically, only 1 out of 1,000 randomized codes resulted in a greater overlap for QGRSmapper, and none for the other two prediction tools (Fig. 5*A*). Interestingly, the number of predicted rG4s in the CDS of the recoded mRNA sequences was in general considerably lower than for the native sequences, which could in turn lead to a lower number of RGG motifs partly encoded by rG4s simply because of their lower abundance. However, the percentage of rG4s that code for RGG motifs is still significantly higher for the native sequences, showing that predicted rG4s in the native sequences have a higher propensity to overlap with RGG motifs in addition to being more abundant. This suggests that the observed effect does not solely depend on the number of predicted rG4s, but also on the probability of rG4 sequences to overlap with RGG-motif coding sequences (*SI Appendix*, Fig. S7).

Longer RGG-motif coding sequences naturally have a higher chance of coinciding with some rG4 sequence at random, simply because they cover larger regions. Nevertheless, although generally higher, the *P*-values reach significant levels regardless of RGG-motif length, for all experimental and computational methods and all randomized backgrounds (Fig. 5*B*). This suggests that the increase in the frequency of RGG motifs encoded by rG4s with increasing motif length (Fig. 3*C*) cannot be fully explained by their length alone, but follows significantly from the structure of the genetic code. In fact, considering that glycine is encoded by GGN, a protein sequence that contains 4 glycines in close proximity to each other is sufficient to ensure that at least a theoretical noncanonical rG4 sequence (containing at least 2 G-quartets) is present in the respective mRNA CDS.

### Examples of RNA-Binding Proteins with Potential rG4/RGG Coding–Binding Relationship.

**Fig. 6.**
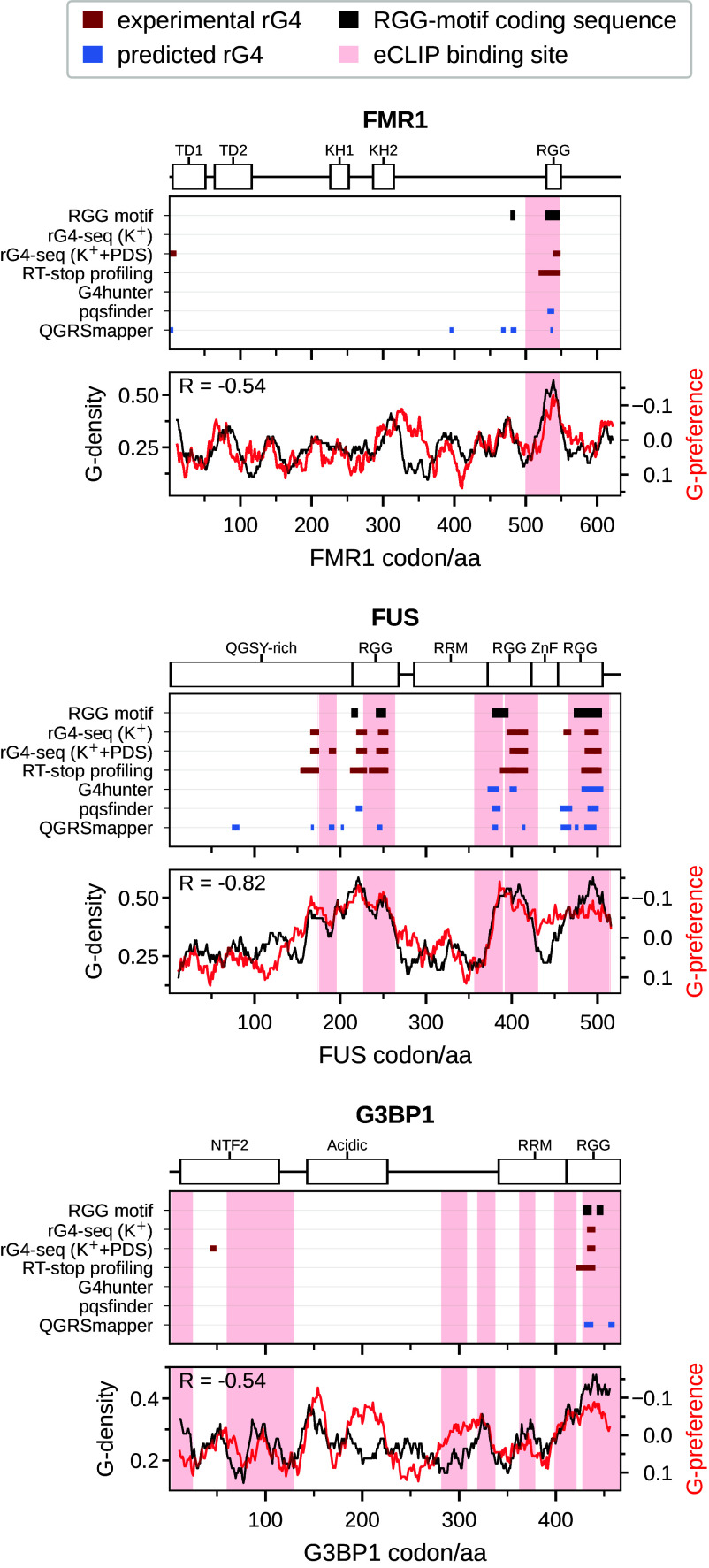
eCLIP-detected interactions sites overlap with RGG motifs encoded by rG4s for several RBPs. Location of the experimentally determined (red) and predicted (blue) rG4s in the CDSs of FMRP, FUS, and G3BP1, together with the location of RGG-coding regions (black) and the known RNA-binding domains (above). Regions of eCLIP-detected autogenous binding are highlighted in light red. In the *Bottom* row, CDS guanine-density profiles are depicted together with the corresponding protein guanine-preference (56) profiles. Pearson correlation coefficients between the profiles are given in the *Top Left* corners. The two different y-axes are oriented in opposite directions for visualization, with negative Pearson R values indicating profile matching, reflecting the standard thermodynamic convention for expressing binding affinities.

As mentioned above, FMRP interacts with its autogenous mRNA via an RGG motif that binds to an rG4 that encodes it (59, 61, 62). Consistent with this finding, we have identified rG4 sequences in the RGG-motif coding region of FMRP in experimental rG4-seq (K^+^ + PDS) and RT-stop profiling datasets as well *pqsfinder* and *QGRSmapper* predicted datasets (Fig. 6). Additionally, eCLIP data available from POSTAR3 (76) reveals a binding site of FMRP in its own CDS precisely at that location, further agreeing with the known autogenous interaction between FMRP and its mRNA. The complementarity hypothesis proposes that proteins in general tend to interact with their mRNA CDS in a coaligned fashion (55). Specifically, this could take place in select regions of protein and RNA sequences with extreme compositional features. In fact, in the case of FMRP, such binding is realized in a CDS region characterized by a strong peak in guanine density, where the corresponding protein sequence peaks in guanine-preference as evaluated using a knowledge-based affinity scale derived by the analysis of 3D structures of RNA–protein complexes (56) (Fig. 6). Interestingly, a similar situation is observed in the case of FUS and G3BP1, both of which are known to interact with rG4s via their RGG domains (26, 28, 34). Specifically, both proteins contain RGG motifs that are partly encoded by rG4s, as identified by all three experimental methods and at least one rG4 prediction tool (Fig. 6). Furthermore, like in the case of FMRP, eCLIP data indicate autogenous binding in the regions where RGG-motif coding sequences and rG4s overlap. Most notably, in the case of FUS, three clusters of rG4 sequences and eCLIP binding sites can be observed precisely at the location of three RGG-motif coding sequences, in addition to exhibiting strong matching between protein guanine-preference and mRNA guanine-density profiles in these regions (Fig. 6). Although the eCLIP binding sites in the CDS of these proteins do not provide information on how the interaction is realized, this observation suggests that like FMRP, those proteins could bind with their RGG motif(s) to rG4s in their own mRNA, precisely at the location where the corresponding RGG motif is encoded.

**Fig. 7.**
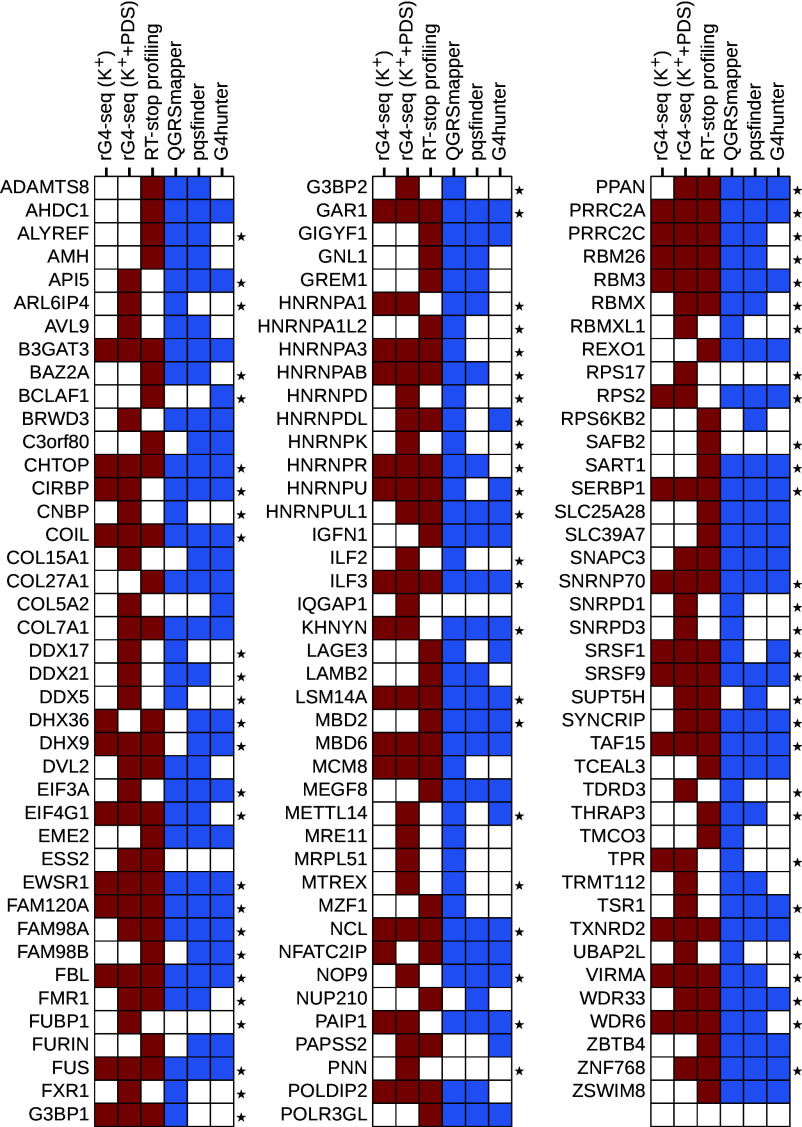
Human proteins that contain RGG motifs encoded by experimental rG4s. Proteins containing an RGG motif that is partly encoded by an rG4 as detected by at least one experimental method analyzed. Red and blue colors indicate that a given protein contains an RGG motif that is partly encoded by an rG4 of the corresponding experimental or computational dataset, respectively. Proteins that are annotated with the GO term *RNA binding* are marked with a star.

At the moment, such strong support regarding both the coding relationship between RGG motifs and rG4s as well as autogenous binding as observed via eCLIP exist for FMRP, FUS, and G3BP1 only. Furthermore, just like FMRP, FUS is also known to exhibit autoregulatory behavior (77, 78). On the other hand, we identify 743 proteins with at least one RGG-motif coding sequence that coincides with an rG4 in one of the datasets investigated (*SI Appendix*, Fig. S8), with 122 of those having an RGG motif that coincides with an experimentally determined rG4 (Fig. 7). While not all these interactions may be realized, our results hint at the possibility that such a connection exists for many proteins, especially for longer RGG motifs in RBPs. As a case in point, for all proteins that are known to interact with rG4s via their RGG motifs, mentioned in the introduction, we found that these motifs are encoded by rG4s as assigned consistently by both experimental and computational methods (Fig. 7).

## Discussion

The RGG motif is the second-most abundant RNA-binding motif in proteins (24, 25) and rG4 is one of its best-characterized and most ubiquitous targets (18, 31). The present study provides a quantitative characterization of the intrinsic connection between the rG4/RGG recognition and the structure of the genetic code, as a potentially powerful, yet mechanistically simple contribution to autogenous mRNA/protein interactions and the associated autoregulatory feedback loops. In particular, our analysis identifies hundreds of rG4/RGG pairs related by coding and, as such, points to a high intrinsic potential for these motifs to interact in an autogenous context under different circumstances. While other RNA binding domains, including RRM and zinc finger domains (14, 17, 18), also interact with rG4s, they are frequently found in RBPs in combination with RGG domains and are known to enhance their binding activity synergistically (26, 34, 79). It has also been proposed that the flanking regions of RGG motifs may modulate their specificity to achieve a more promiscuous binding mode (80). An exciting area of future work concerns the role of rG4-encoded RGG motifs in such multivalent binding schemes.

Our analysis shows that RNA binding is significantly enriched among proteins which contain RGG motifs encoded by experimentally determined rG4s as compared to all RGG-containing proteins, with as much as 68 different RBPs in the former category for the rG4-seq (K^+^ + PDS) dataset (Fig. 4). This can be attributed to the fact that RBPs tend to exhibit longer RGG motifs (*SI Appendix*, Fig. S9), which in turn increases the chances of observing a relevant overlap with an rG4 region in its CDS, especially in the case of experimentally determined rG4s (Fig. 3*C*). However, although length alone contributes to an increased probability to obtain an overlap, the structure of the genetic code itself has a significant influence in this regard (Fig. 5*B*). Therefore, it is possible that the high enrichment of RBPs in this case indeed reflects the known ability of many RBPs to influence different aspects of their own mRNA’s life cycle. As regulators involved in all aspects of RNA biology, including transcription, splicing, translation, and decay (81, 82), RBPs themselves must be tightly controlled and autoregulation presents one of the most robust paradigms in this regard (41, 54). Importantly, the fact that our GO analysis results almost exclusively in a group of proteins that are known to heavily undergo autogenous interactions (54) supports the possibility that many RGG motifs and rG4s that are related by coding may indeed also undergo binding.

The rG4/RGG interactions are generally associated with different biological functions (1–3, 14). Given that rG4s in coding sequences typically act as roadblocks for ribosomes and cause translational pausing, stalling, or frameshifting (2, 83), many of these functions are related to translation regulation. For example, the RGG motif of Aven interacts with rG4s in the coding sequences of MLL1 and MLL4 and recruits DHX36, likely unwinding the rG4 structures and promoting their translation (63). On the other hand, FMRP binds to an rG4 in the coding sequence of amyloid precursor protein (APP), inhibiting its translation (2, 84). Interestingly, while FMRP binding to its own mRNA has been reported to have no impact on translation (62), it has been shown to autoregulate its alternative splicing (61, 62). Considering these different examples, we expect that the autogenous rG4/RGG binding discussed herein may also result in diverse autoregulatory functions and mechanisms, depending on the specific protein, cellular context, or interplay with other regulators.

The present results also provide a possible mechanism of how the formation of membraneless biomolecular condensates (85) in the cell could in part be regulated. Namely, it is known that RGG motifs often moonlight as stickers in protein–protein interactions that underlie the liquid–liquid phase separation processes (27). In addition, it is known that RNA can significantly affect the critical concentration for phase separation of many proteins by modulating their interactions or serving as a scaffold (86, 87). It is possible that the autogenous RGG/rG4 interactions discussed herein play a direct role in this. For example, the three RBPs discussed directly in this study, FUS, FMRP, and G3BP1, are all known to form biomolecular condensates (88–90), as well as interact with their own mRNAs. It would be exciting to explore the possible effect that a direct competition between protein–protein RGG/RGG and RNA–protein rG4/RGG interactions may have on condensate formation in these cases. This becomes particularly pertinent if one considers the emerging roles of rG4s in the formation of stress granules (12, 13, 91) and the involvement of the above-mentioned proteins, and particularly G3BP1, as key players in the process (27, 90–92).

**Fig. 8.**
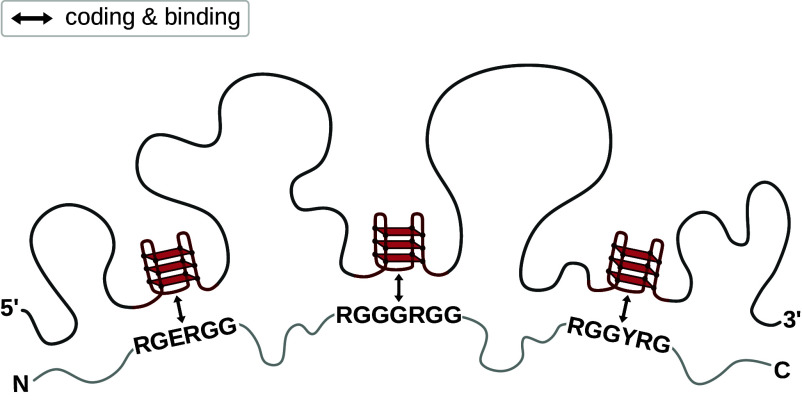
Autogenous interaction between RGG motifs and rG4s. Binding between an RGG motif in a protein and an rG4 that encodes this motif in the protein’s own mRNA may contribute to autoregulatory behavior. Occurrence of multiple such interactions within the same mRNA/protein pair, as depicted here, may additionally impart a coaligned orientation to the two biomolecules.

Furthermore, the present analysis also provides a mechanistic context for thinking about the mRNA/protein complementarity hypothesis, the idea that autogenous mRNA/protein pairs may in general exhibit a tendency to interact, especially if unstructured, reflecting the driving forces behind the origin of the universal genetic code (54–58). Importantly, the hypothesis proposes that mRNAs interact with their autogenous proteins in a coaligned manner, with contact points between the two partners being related by the coding relationship. A challenge to this proposal concerns the fact that the contour length of a typical mRNA coding region is approximately 4.5 times longer than the contour length of the polypeptide encoded by it. The proposed autogenous RGG/rG4 interaction mode provides a simple solution to this challenge, at least for some sequences, in that a typical RGG motif is largely unstructured and extended, while a typical rG4 is extremely compact. For example, the radius of gyration of the 35-nucleotide sc1 rG4 is only 1.4 nm, approximately 6 times more compact than an elongated 35-nucleotide long RNA molecule. Such a distance can in principle be traversed by an extended peptide of only several amino acids. This example illustrates that physically and geometrically realistic, well-characterized binding modes, such as that between RGG motif and rG4, can be consistent with the complementarity hypothesis despite the major incongruence between the lengths of mRNAs and their autogenous proteins, as illustrated in Fig. 8. Additionally, the complementarity hypothesis does not require autogenous mRNA/protein pairs to directly interact along their full lengths: while the compositional biases present in complete sequences may contribute to the colocalization and alignment of the two binding partners, the ultimate contact points could be established between select anchor points only (Fig. 8). Note that coaligned binding need not be a prerequisite for autoregulation.

Although the SELEX-designed sc1 rG4 does not code for the FMRP RGG motif, its high-resolution structure (40) provides several relevant points in this regard. First, in this example, the majority of the rG4 nucleotides do not directly interact with the FMRP RGG motif (Fig. 1*A*), but rather the binding takes place primarily at select spots in the quadruplex/duplex junction. In the case of larger interacting domains or complete polymers, such binding spots may be distributed over even larger stretches, while still agreeing with and reflecting the coding relationship between the bound fragments. Second, in the case of the sc1 rG4/FMRP RGG complex, the two arginine residues in the RGG motif make direct contacts with guanine nucleotides of two arginine codons CGC and CGG in the sc1 structure (*SI Appendix*, Fig. S10). Despite how suggestive and illustrative of the complementarity hypothesis this example is, we do not expect that the residues in RGG motifs necessarily always directly bind to their autogenous codons in rG4s—rather, a more dynamic, fluid binding mode is expected (55). Moreover, the individual preferences between nucleobases and amino acids could be important for nonautogenous interactions as well. Specifically, while the observed interactions between rG4s and RGG motifs (26, 34, 38) may not be related by a direct coding relationship, the compositional biases present within these domains could contribute to the tendency of them to interact in general.

Our analysis has identified FMRP, FUS, and G3BP1 as three well-known RBPs in which an rG4/RGG pair connected by coding overlaps with the known binding sites as identified by eCLIP (Fig. 6). Of all such examples, FUS protein represents arguably the strongest, not-yet characterized candidate for strong autogenous interactions based on the rG4/RGG binding mode. As shown in Fig. 6, three separate RGG motifs in FUS are encoded by different rG4-containing regions, as identified both computationally and experimentally, with eCLIP data showing strong signatures of binding precisely in the regions of rG4/RGG co-occurrence. What is more, due to these same reasons, FUS is arguably the best test system for analyzing the predictions of the complementarity hypothesis concerning coaligned binding. If the three RGG motifs indeed interact directly with the respective rG4 motifs that encode them, this would create a correct register for the remainder of the FUS protein sequence to interact with its mRNA in a coaligned manner (Fig. 8). As a consequence, other RNA-binding domains in FUS, i.e., RRM and Zn-finger, will also have an increased chance to bind to their respective coding sequences. Here, it should be noted that the agreement with eCLIP data does not unequivocally prove that rG4/RGG pairs related by coding indeed interact—for this, a detailed biophysical evidence is required, as obtained in the case of FMRP (61).

Here, we have focused on the coding relationship between RGG motifs and rG4s, but there are other amino acid repeats encoded by rG4 sequences, frequently including, for example, glutamate or alanine (*SI Appendix*, Fig. S11). Interestingly, glutamate exhibits the highest predicted binding affinity to guanine according to knowledge-based nucleobase/amino acid affinity scales (56), while rG4-binding proteins have previously been shown to also be enriched in glutamate as compared to random proteins (23). Moreover, a high frequency of RGG-motif-related sequences encoded by rG4 sequences such as RGG, GGG, or GRG tripeptides (*SI Appendix*, Fig. S11), which are often not identified as being a part of RGG motifs by the definition employed herein, suggests that this definition may have been too stringent. However, for consistency and to avoid biases, it was critical that we adopt a widely accepted definition of RGG proposed by others (24).

Finally, it is interesting to speculate whether the coding/binding relationship, as discussed here for rG4/RGG pairs, also exists for other well-known RNA-binding domains such as RRMs, KH domains, or Zn-fingers. A possible strategy for addressing this question may be to systematically analyze the known RNA motifs that these domains bind to and study their potential enrichment in the corresponding CDS regions of autogenous mRNAs. This work is under way and will be reported elsewhere. Overall, the present study traces out a framework for future work aimed toward providing a mechanistic understanding of autoregulatory feedback loops in the cell and highlights the centrality of the genetic code in cellular organization.

## Supplementary Material

Appendix 01 (PDF)

## Data Availability

Scripts for analysis and auxiliary data have been deposited in Zenodo (93).
