# Supplementary material for: Coding relationship links RNA G-quadruplexes and protein RGG motifs in RNA-binding protein autoregulation
Source: Proc Natl Acad Sci U S A. 2025 Jan 23;122(4):e2413721122. doi: 10.1073/pnas.2413721122 (PMC11789052; doi:10.1073/pnas.2413721122)
Supplement: Supplementary file 1 — Appendix 01 (PDF) [file pnas.2413721122.sapp.pdf]

PNAS

Supporting Information for

Coding relationship links RNA G-quadruplexes and protein RGG motifs in RNA-binding protein autoregulation

Marlene Adlhart, Daniel Hoffmann, Anton A. Polyansky & Bojan Žagrović*

***Correspondence:**

Email: bojan.zagrovic@univie.ac.at

Tel: +43 2 4277 52271

Fax: +43 1 4277 9522

This PDF file includes:

Figures S1 to S11

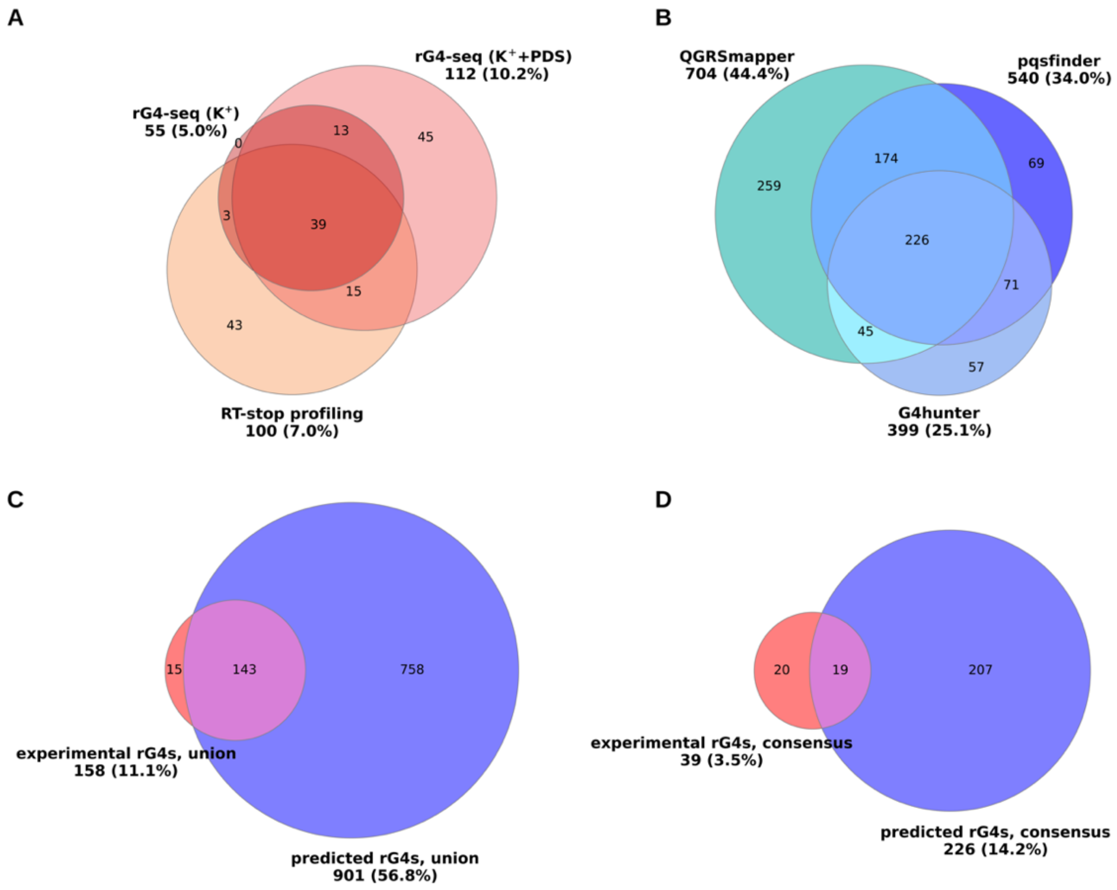

Figure S1. Comparison of RGG motifs identified as being partly encoded by rG4s as determined by different methods. A) Overlap between RGG motifs partly encoded by experimentally determined and B) computationally predicted rG4s. C) Overlap between RGG motifs partly encoded by an rG4 of at least one experimental dataset and an rG4 identified by at least one prediction tool. D) Overlap between RGG motifs that are identified as being partly encoded by all three experimental datasets and all three prediction tools.

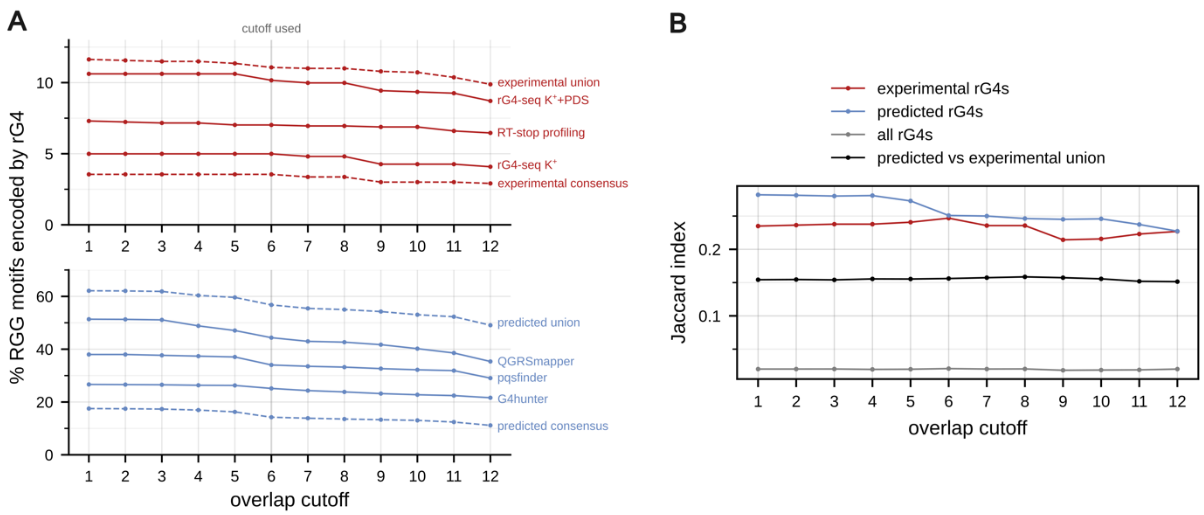

Figure S2. Analysis of overlap cutoff used to define RGG motifs encoded by rG4s. A) Percentage of RGG motifs partly encoded by rG4s, depending on the overlap cutoff used for the different experimental and predicted rG4 datasets. B) Jaccard indices, calculated as the size of the intersection divided by the size of the union, between the sets of RGG motifs identified as being partly encoded by different rG4 identification methods, depending on the overlap cutoff. Shown are Jaccard indices for the overlap between the three experimental datasets, the three predicted datasets, all rG4 datasets combined, as well as for the overlap between predicted and experimental unions.

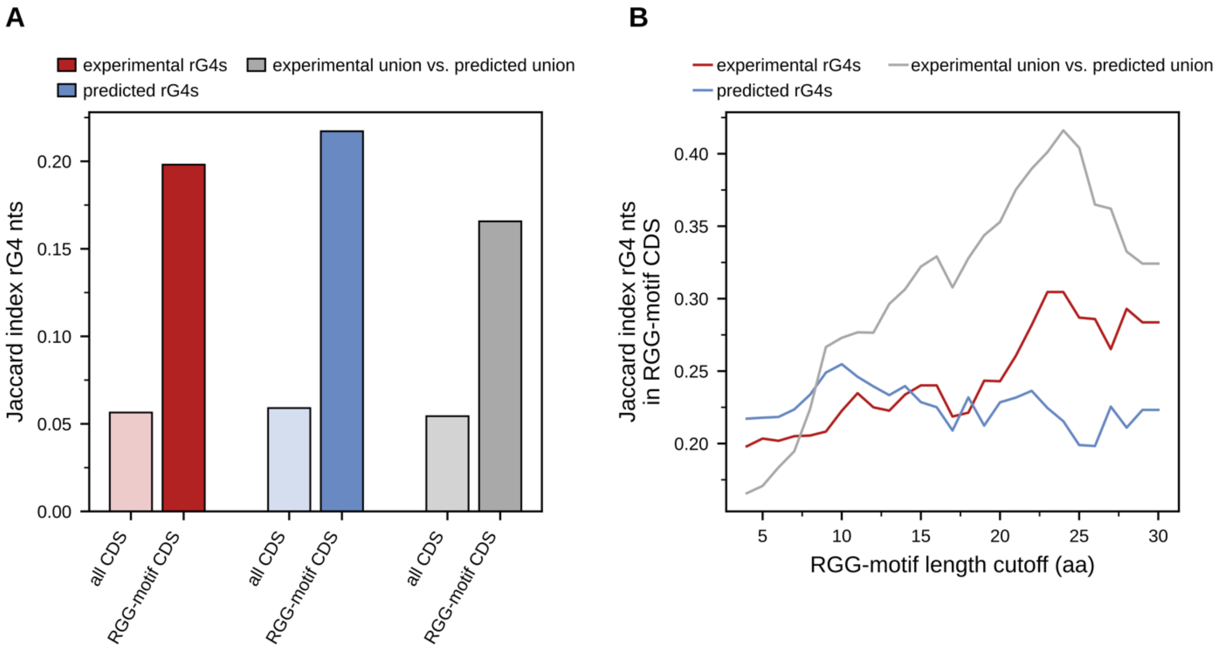

Figure S3. Overlap between rG4 sequences identified in different experimental and computational datasets is greater within RGG-motif coding sequences. A) Jaccard indices, calculated as the size of the intersection divided by the size of the union, between the sets of nucleotides that belong to rG4 sequences in the three experimental rG4 datasets (red) and the three computational rG4 datasets (blue), as well as between the sets of nucleotides that belong to rG4 sequences of at least one experimental or at least one predicted rG4 sequence (grey). Shown are Jaccard indices for the overlap between rG4 sequences in all CDS regions of MANE SELECT transcripts, or for the overlap of rG4 sequences in RGG-motif coding sequences only. B) Jaccard index between the nucleotides that belong to rG4 sequences in RGG-motif coding sequences, depending on RGG-motif length cutoff used to define RGG regions.

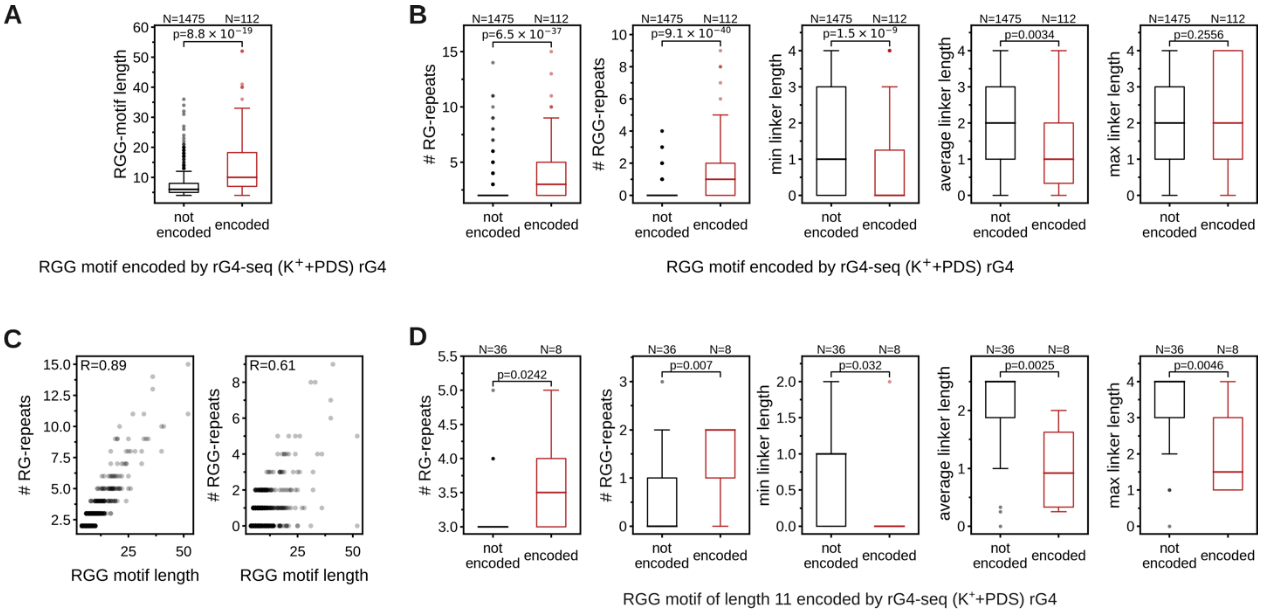

E

	rG4-seq					
	K+	K+PDS	RT-stop profiling	QGRSmapper	pqsfinder	G4hunter
RGG-motif length	8.24	6.82	4.61	1.68	1.74	2.14
# RG-repeats	2.20	1.83	1.36	0.55	0.50	0.63
# RGG-repeats	1.88	1.41	1.15	0.41	0.33	0.38
min linker length	-0.89	-0.81	-0.91	-0.55	-0.37	-0.36
average linker length	-0.51	-0.42	-0.65	-0.39	-0.21	-0.21
max linker length	0.14	0.16	-0.21	-0.18	0.00	0.00

F

	rG4-seq					
	K+	K+PDS	RT-stop profiling	QGRSmapper	pqsfinder	G4hunter
	-	-	-	-	-	
	0.68	0.46	0.52	0.24	0.16	0.07
	0.87	0.92	0.87	0.89	0.49	0.22
	-0.38	-0.50	-0.55	-0.48	-0.14	-0.02
	-1.20	-0.97	-1.03	-0.67	-0.42	-0.19
	-1.84	-1.33	-1.37	-0.88	-0.67	-0.34

G

	average linker composition			
	encoded	not encoded	Δ	p
A	6.5	8.3	-1.8	7.16E-01
C	0.1	2.2	-2.0	5.20E-02
D	6.6	4.7	1.9	3.65E-02
E	2.5	6.7	-4.2	2.68E-02
F	10.5	3.5	7.1	8.20E-09
G	23.6	9.3	14.3	6.08E-10
H	1.4	3.1	-1.6	1.54E-01
I	1.6	2.6	-1.0	7.66E-01
K	2.0	4.0	-1.9	5.84E-02
L	2.5	8.3	-5.8	4.18E-02
M	3.8	1.0	2.8	3.37E-05
N	3.2	2.4	0.9	2.24E-01
P	10.3	11.0	-0.7	9.30E-01
Q	1.3	3.5	-2.1	2.00E-01
R	6.1	9.6	-3.6	6.00E-01
S	9.1	10.4	-1.2	3.12E-01
T	3.1	3.3	-0.3	2.91E-01
V	1.5	3.5	-1.9	3.53E-01
W	0.9	1.4	-0.5	9.04E-01
Y	3.2	1.5	1.7	1.20E-02

H

	rG4-seq					
	K+	K+PDS	RT-stop profiling	QGRSmapper	pqsfinder	G4hunter
A	-2.0	-1.8	-2.6	0.2	4.0	0.0
C	-1.8	-2.0	-1.0	-1.3	-1.1	-1.1
D	2.5	1.9	0.8	0.1	-0.3	-0.5
E	-5.1	-4.2	-3.4	-2.4	-0.1	1.4
F	7.6	7.1	2.2	-0.7	-2.4	-0.2
G	20.3	14.3	21.5	10.0	8.9	8.7
H	0.0	-1.6	-0.6	-1.3	-1.9	-1.1
I	0.3	-1.0	-1.4	-1.4	-2.5	-2.1
K	-1.3	-1.9	-3.3	-1.5	-1.0	-0.6
L	-6.6	-5.8	-3.6	-3.0	-1.5	-0.6
M	2.3	2.8	1.4	0.2	-0.6	-0.2
N	2.9	0.9	0.1	0.3	-2.3	-2.5
P	-6.0	-0.7	-3.9	-3.2	-2.2	-2.4
Q	-2.7	-2.1	-0.4	-0.2	0.3	0.5
R	-6.3	-3.6	-3.2	5.0	3.2	-1.0
S	-4.9	-1.2	-3.8	-0.9	0.5	0.2
T	-2.1	-0.3	-0.4	0.0	-1.1	0.1
V	-2.7	-1.9	-0.4	-1.1	0.0	0.0
W	0.5	-0.5	-0.5	1.9	0.6	1.7
Y	5.0	1.7	2.4	-0.8	-0.6	-0.4

Figure S4. RGG motifs partly encoded by rG4s preferentially contain long, compact RG/RGG repeats and glycine-rich linker sequences. A) Distribution of RGG-motif lengths for RGG motifs not encoded (black) or partly encoded by rG4s detected via rG4-seq (K^++PDS). B) Distribution of the number of RG (including RGG) or RGG repeats along with distributions of the minimum, average and maximum linker length in RGG motifs not encoded (black) or partly encoded (red) by rG4s detected via rG4-seq (K^++PDS). P-values are calculated using the two-sided Mann-Whitney-U-test. C) Correlation between the number of RG or RGG repeats and RGG-motif length. Pearson correlation coefficient is given in the top left corner. D) Same as in B) but including only RGG motifs of fixed length equal to 11. E) Differences in average RGG-

motif length, number of RG or RGG repeats and minimum, average and maximum linker length for RGG motifs partly encoded vs. not encoded by rG4s, for different experimental and computational rG4 datasets used. Grey shading indicates no significant difference ($p \geq 0.05$) between the two distributions, based on two-sided Mann-Whitney-U-test. F) Same as in E) but including only RGG motifs of fixed length equal to 11 in the analysis. G) Average amino-acid composition of linker sequences for RGG motifs not encoded or partly encoded by rG4s detected via rG4-seq (K^++PDS), as well as their difference. P-values are calculated using the two-sided Mann-Whitney-U-test. Amino acids associated with a p-value < 0.05 are highlighted with a border. H) Differences in the average linker amino-acid composition for RGG motifs not encoded or partly encoded by rG4s, for the different experimental and computational rG4 datasets. Grey shading indicates no significant difference ($p \geq 0.05$) between the two distributions (two-sided Mann-Whitney-U-test).

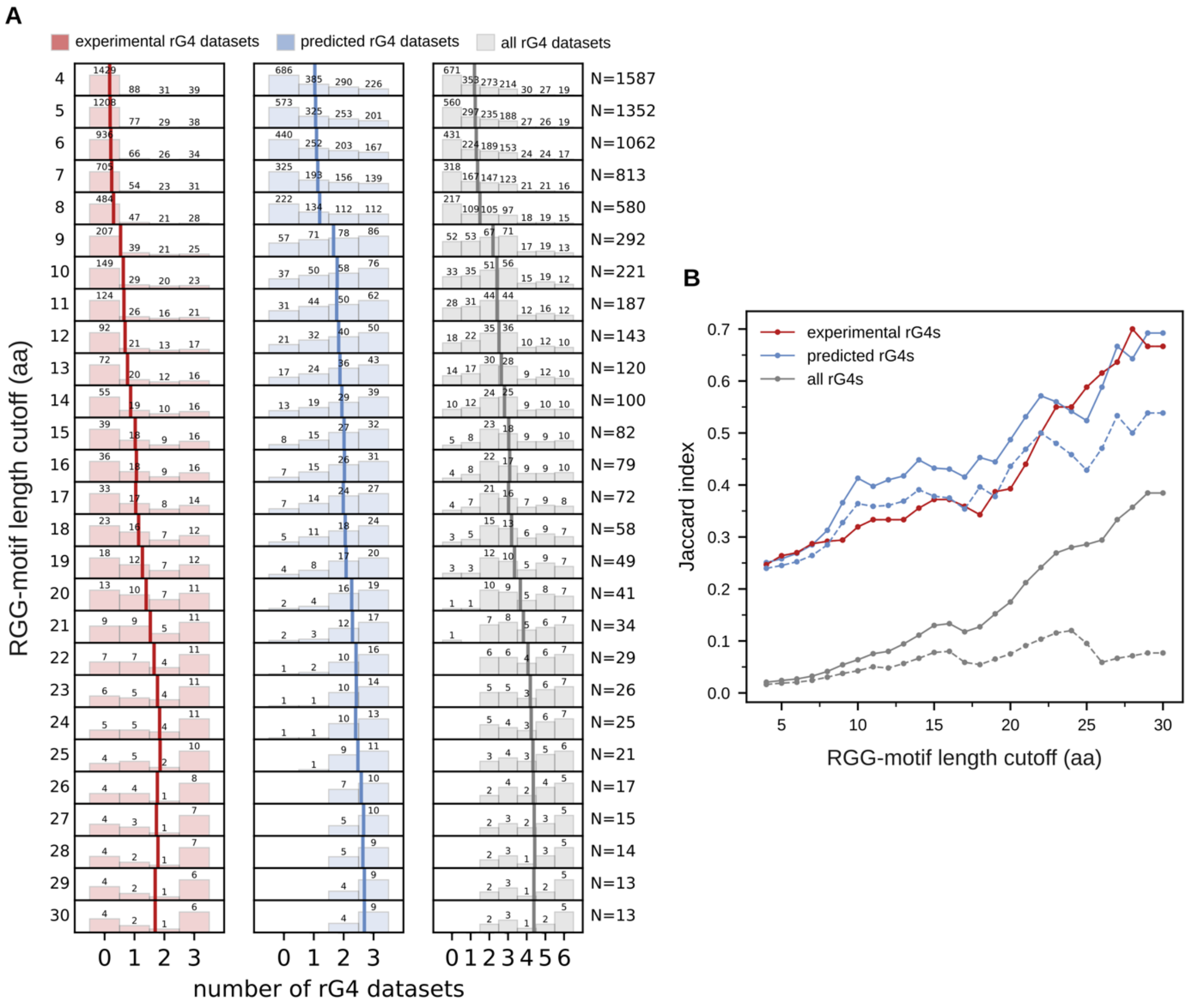

Figure S5. rG4/RGG coding relationship detection is a function of RGG-motif length. A) Distribution of the number of experimental (red), computational (blue) and experimental + computational (grey) rG4 datasets that identify an RGG motif as being partly encoded by an rG4, for different RGG-motif length cutoffs. Solid line corresponds to the average number of datasets. Individual values on top of the bars refer to the number of RGG motifs that are identified as being partly encoded in the corresponding number of rG4 datasets. The number of RGG motifs with a length greater or equal to the motif-length cutoff is given on the right. B) Jaccard indices, calculated as the size of the intersection divided by the size of the union, between the sets of RGG motifs that are identified as being partly encoded by an rG4 for experimental (red), computational (blue), and experimental + computational (grey) methods, depending on the motif-length cutoff. Jaccard indices between the sets of RGG motifs, which are identified as being partly encoded by different rG4s at the exact same location within the motif, are indicated with a dashed line.

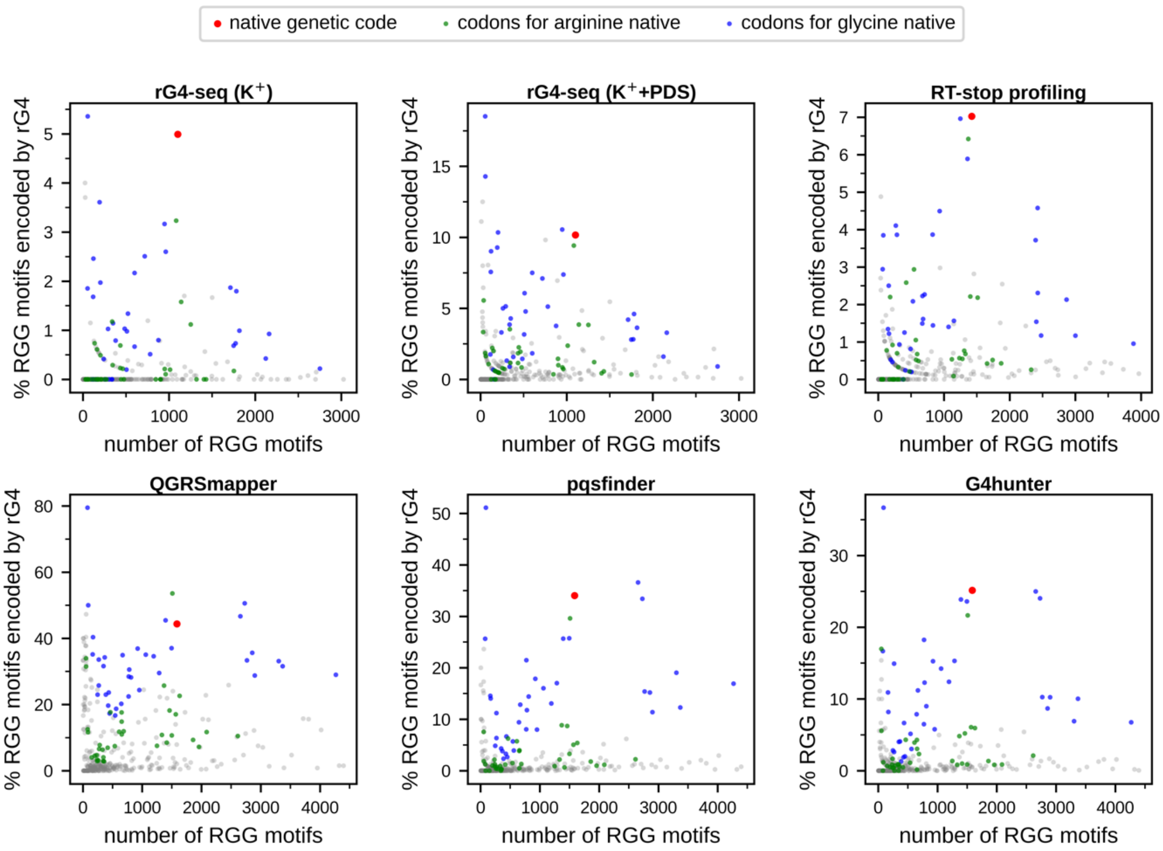

Figure S6. rG4/RGG coding relationship is a consequence of the structure of the standard genetic code. Percentage of RGG motifs that are encoded by rG4s depending on the number of RGG motifs in translations obtained from alternative genetic codes generated by reassigning arginine and glycine to different codon boxes. Datapoints depicted in blue represent the generated codes where the codons for glycine remained native, while datapoints depicted in green represent codes where codons for arginine remained native. Datapoints depicted in red correspond to the native genetic code.

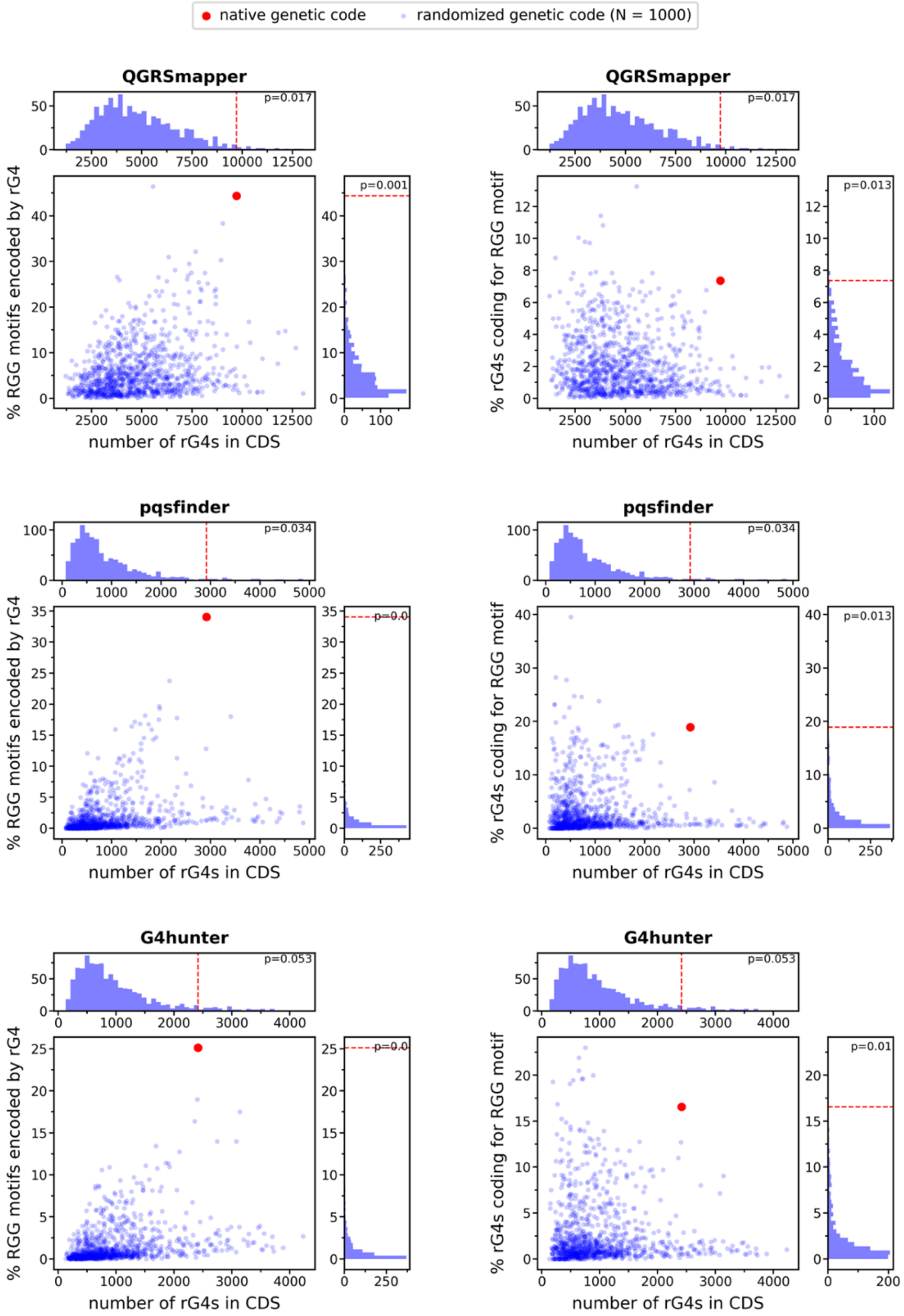

Figure S7. Randomization analysis for computationally predicted rG4s. Percentage of RGG motifs partly encoded by rG4s (left column) and percentage of rG4s coding for RGG motifs (right column), as a function of the number of rG4s predicted in the CDS of proteins containing RGG motifs for recoded mRNA sequences, for the three different prediction tools. The values obtained for the native genetic code are highlighted in red.

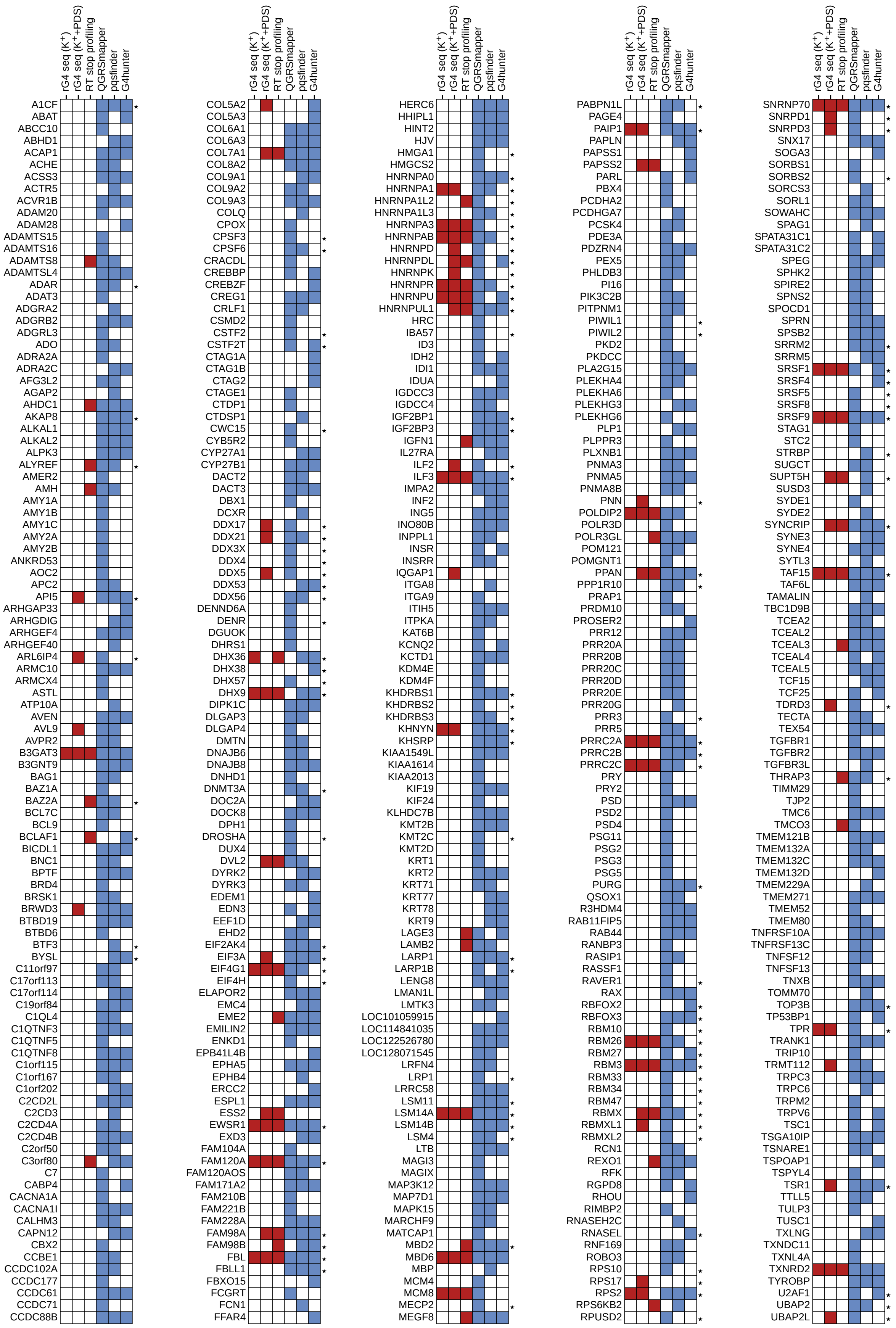
rG4 seq (K$^+$)
rG4 seq (K$^+$+PDS)
RT stop profiling
QGRSmapper
pqsfinder
G4hunter
A1CF ABAT ABCC10 ABHD1 ACAP1 ACHE ACSS3 ACTR5 ACVR1B ADAM20 ADAM28 ADAMTS15 ADAMTS16 ADAMTS8 ADAMTSL4 ADAR ADAT3 ADGRA2 ADGRB2 ADGRL3 ADO ADRA2A ADRA2C AFG3L2 AGAP2 AHDC1 AKAP8 ALKAL1 ALKAL2 ALPK3 ALYREF AMER2 AMH AMY1A AMY1B AMY1C AMY2A AMY2B ANKRD53 AOC2 APC2 API5 ARHGAP33 ARHGDIG ARHGEF4 ARHGEF40 ARL6IP4 ARMC10 ARMCX4 ASTL ATP10A AVEN AVL9 AVPR2 B3GAT3 B3GNT9 BAG1 BAZ1A BAZ2A BCL7C BCL9 BCLAF1 BICDL1 BNC1 BPTF BRD4 BRSK1 BRWD3 BTBD19 BTBD6 BTF3 BYSL C11orf97 C17orf113 C17orf114 C19orf84 C1QL4 C1QTNF3 C1QTNF5 C1QTNF8 C1orf115 C1orf167 C1orf202 C2CD2L C2CD3 C2CD4A C2CD4B C2orf50 C3orf80 C7 CABP4 CACNA1A CACNA1I CALHM3 CAPN12 CBX2 CCBE1 CCDC102A CCDC177 CCDC61 CCDC71 CCDC88B
COL5A2 COL5A3 COL6A1 COL6A3 COL7A1 COL8A2 COL9A1 COL9A2 COL9A3 COLQ CPOX CPSF3 CPSF6 CRACDL CREBBP CREBZF CREG1 CRLF1 CSMD2 CSTF2 CSTF2T CTAG1A CTAG1B CTAG2 CTAGE1 CTDP1 CTDSP1 CWC15 CYB5R2 CYP27A1 CYP27B1 DACT2 DACT3 DBX1 DCXR DDX17 DDX21 DDX3X DDX4 DDX5 DDX53 DDX56 DENND6A DENR DGUOK DHRS1 DHX36 DHX38 DHX57 DHX9 DIPK1C DLGAP3 DLGAP4 DMTN DNAJB6 DNAJB8 DNHD1 DNMT3A DOC2A DOCK8 DPH1 DROSHA DUX4 DVL2 DYRK2 DYRK3 EDEM1 EDN3 EEF1D EHD2 EIF2AK4 EIF3A EIF4G1 EIF4H ELAPOR2 EMC4 EME2 EMILIN2 ENKD1 EPB41L4B EPHA5 EPHB4 ERCC2 ESPL1 ESS2 EWSR1 EXD3 FAM104A FAM120A FAM120AOS FAM171A2 FAM210B FAM221B FAM228A FAM98A FAM98B FBL FBLL1 FBXO15 FCGRT FCN1 FFAR4
HERC6 HHIPL1 HINT2 HJV HMGA1 HMGCS2 HNRNPA0 HNRNPA1 HNRNPA1L2 HNRNPA1L3 HNRNPA3 HNRNPAB HNRNPD HNRNPDL HNRNPK HNRNPR HNRNPU HNRNPUL1 HRC IBA57 ID3 IDH2 IDI1 IDUA IGDCC3 IGDCC4 IGF2BP1 IGF2BP3 IGFN1 IL27RA ILF2 ILF3 IMPA2 INF2 ING5 INO80B INPPL1 INSR INSRR IQGAP1 ITGA8 ITGA9 ITIH5 ITPKA KAT6B KCNQ2 KCTD1 KDM4E KDM4F KHDRBS1 KHDRBS2 KHDRBS3 KHNYN KHSRP KIAA1549L KIAA1614 KIAA2013 KIF19 KIF24 KLHDC7B KMT2B KMT2C KMT2D KRT1 KRT2 KRT71 KRT77 KRT78 KRT9 LAGE3 LAMB2 LARP1 LARP1B LENG8 LMAN1L LMTK3 LOC101059915 LOC114841035 LOC122526780 LOC128071545 LRFN4 LRP1 LRRC58 LSM11 LSM14A LSM14B LSM4 LTB MAGI3 MAGIX MAP3K12 MAP7D1 MAPK15 MARCHF9 MATCAP1 MBD2 MBD6 MBP MCM4 MCM8 MECP2 MEGF8
PABPN1L PAGE4 PAIP1 PAPLN PAPSS1 PAPSS2 PARL PBX4 PCDHA2 PCDHGA7 PCSK4 PDE3A PDZRN4 PEX5 PHLDB3 PI16 PIK3C2B PITPNM1 PIWIL1 PIWIL2 PKD2 PKDCC PLA2G15 PLEKHA4 PLEKHA6 PLEKHG3 PLEKHG6 PLP1 PLPPR3 PLXNB1 PNMA3 PNMA5 PNMA8B PNN POLDIP2 POLR3D POLR3GL POM121 POMGNT1 PPAN PPP1R10 PRAP1 PRDM10 PROSER2 PRR12 PRR20A PRR20B PRR20C PRR20D PRR20E PRR20G PRR3 PRR5 PRRC2A PRRC2B PRRC2C PRY PRY2 PSD PSD2 PSD4 PSG11 PSG2 PSG3 PSG5 PURG QSOX1 R3HDM4 RAB11FIP5 RAB44 RANBP3 RASIP1 RASSF1 RAVER1 RAX RBFOX2 RBFOX3 RBM10 RBM26 RBM27 RBM3 RBM33 RBM34 RBM47 RBMX RBMXL1 RBMXL2 RCN1 REXO1 RFK RGPD8 RHOU RIMBP2 RNASEH2C RNASEL RNF169 ROBO3 RPS10 RPS17 RPS2 RPS6KB2 RPUSD2
SNRNP70 SNRPD1 SNRPD3 SNX17 SOGA3 SORBS1 SORBS2 SORCS3 SORL1 SOWAHC SPAG1 SPATA31C1 SPATA31C2 SPEG SPHK2 SPIRE2 SPNS2 SPOCD1 SPRN SPSB2 SRRM2 SRRM5 SRSF1 SRSF4 SRSF5 SRSF8 SRSF9 STAG1 STC2 STRBP SUGCT SUPT5H SUSD3 SYDE1 SYDE2 SYNCRIP SYNE3 SYNE4 SYTL3 TAF15 TAF6L TAMALIN TBC1D9B TCEA2 TCEAL2 TCEAL3 TCEAL4 TCEAL5 TCF15 TCF25 TDRD3 TECTA TEX54 TGFBR1 TGFBR2 TGFBR3L THRAP3 TIMM29 TJP2 TMC6 TMCO3 TMEM121B TMEM132A TMEM132C TMEM132D TMEM229A TMEM271 TMEM52 TMEM80 TNFRSF10A TNFRSF13C TNFSF12 TNFSF13 TNXB TOMM70 TOP3B TP53BP1 TPR TRANK1 TRIP10 TRMT112 TRPC3 TRPC6 TRPM2 TRPV6 TSC1 TSGA10IP TSNARE1 TSPOAP1 TSPYL4 TSR1 TTLL5 TULP3 TUSC1 TXLNG TXNDC11 TXNL4A TXNRD2 TYROBP U2AF1 UBAP2 UBAP2L

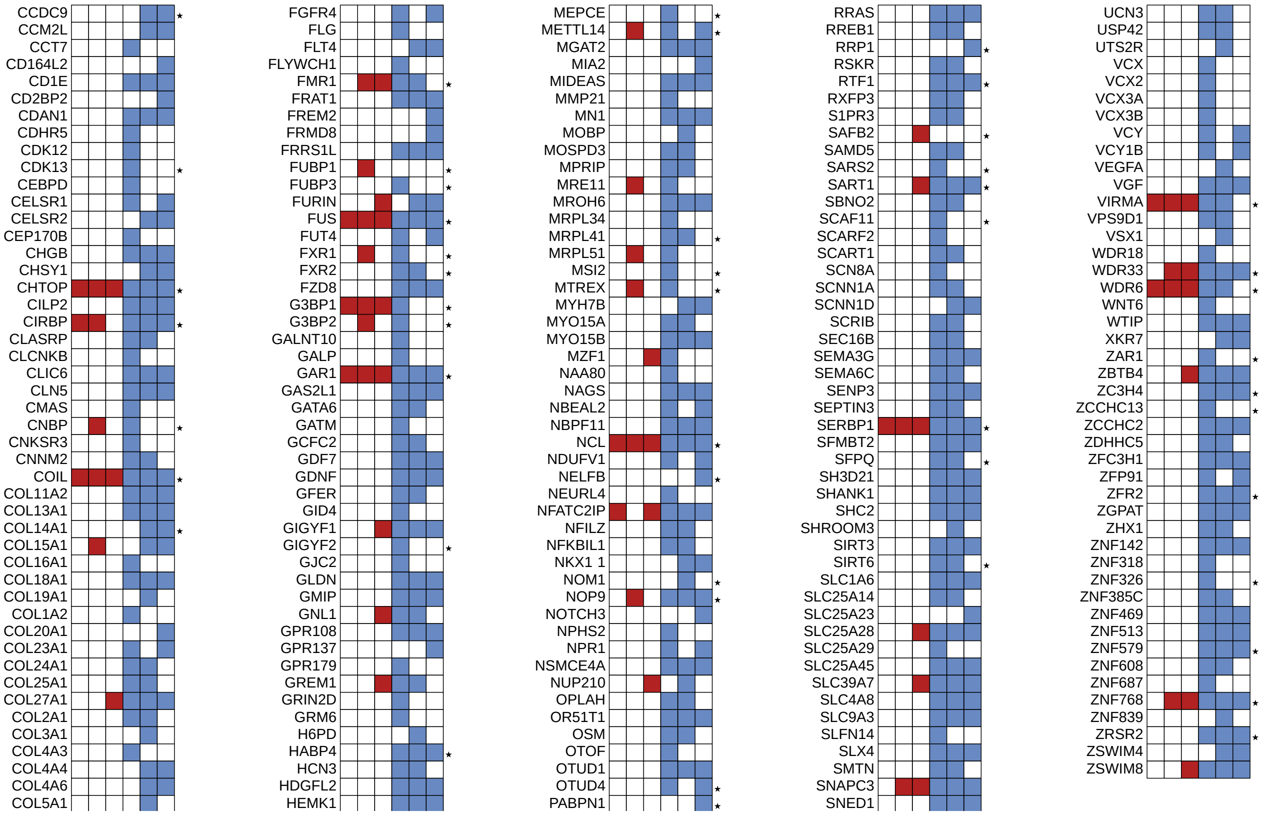

Figure S8. Proteins containing RGG motifs partly encoded by rG4s. All proteins that contain an RGG motif that is partly encoded by an rG4 as detected by one of the analyzed experimental or computational rG4 datasets are listed. Colors indicate that the protein contains an RGG motif that is partly encoded by an rG4 of the corresponding experimental (red) or computational (blue) rG4 dataset. Proteins that are associated with the GO term *RNA binding* are indicated with a star.

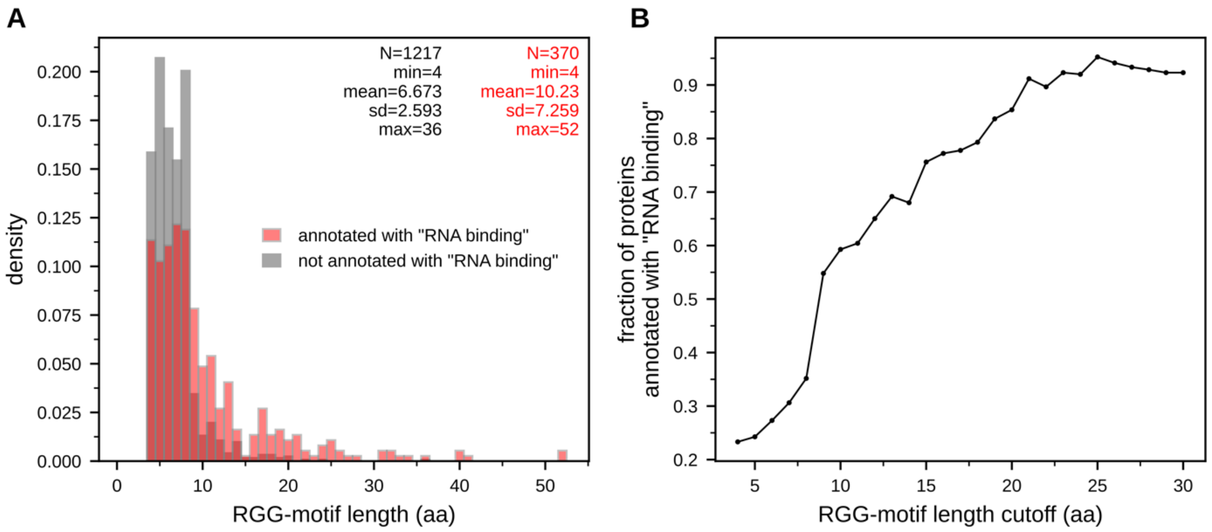

Figure S9. Longer RGG motifs are preferentially found in RBPs. A) Distribution of RGG-motif lengths for proteins annotated (red) or not annotated (grey) with the GO term *RNA-binding*. B) Fraction of RGG-motif containing proteins that are annotated with the GO term *RNA-binding*, depending on the motif-length cutoff.

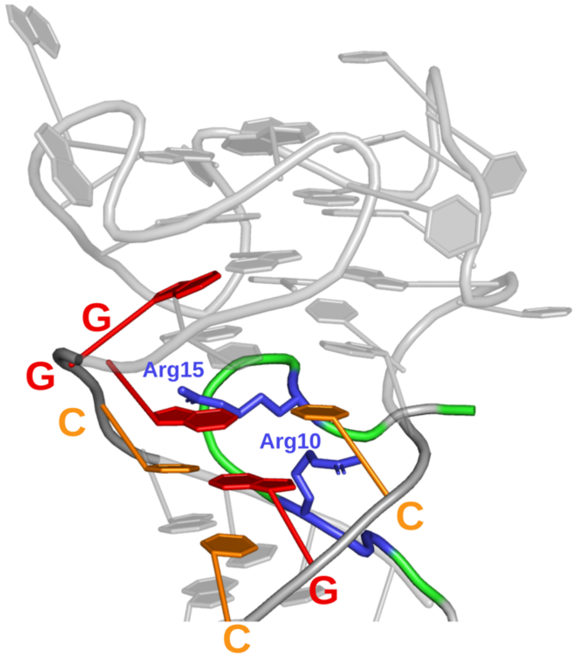

Figure S10. Interaction of FMRP RGG peptide with nucleobases of arginine codons in sc1 rG4. FMRP RGG peptide with sc1 rG4 (PDB 5DE5). The arginine codons in the sc1 rG4 (CGG and CGC) are labeled explicitly. Base-specific interactions are made between Arg10 and Arg15 and the guanines in the arginine codons.

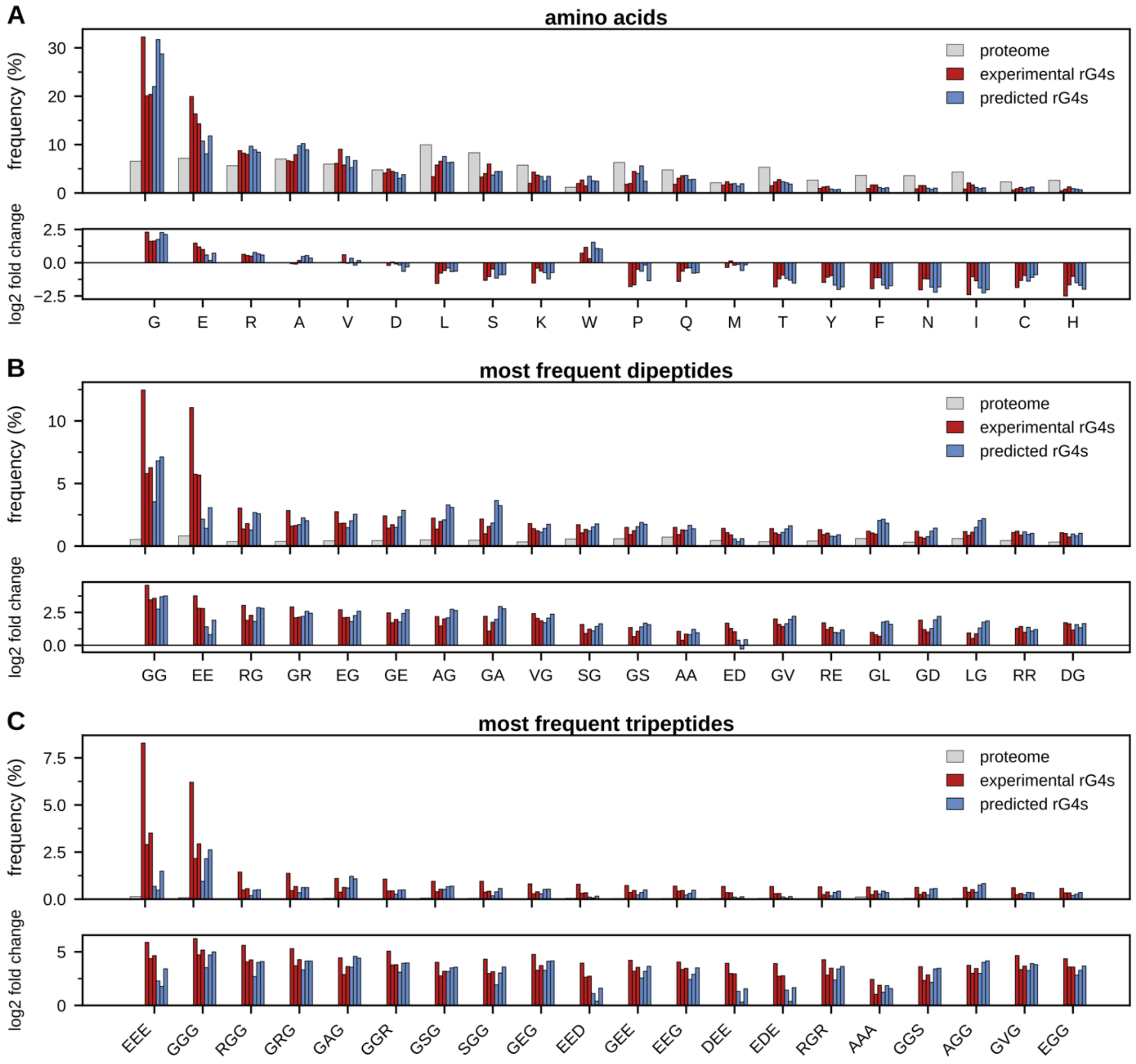

Figure S11. Peptide sequences encoded by rG4s. A) Amino-acid composition of peptide sequences encoded by different experimentally (red) and computationally (blue) derived rG4-sequences (from left to right: rG4-seq (K+), rG4-seq (K++PDS), RT-stop profiling, QGRSmapper, pqsfinder and G4hunter), together with amino-acid composition of all MANE SELECT proteins (grey). Amino acids are sorted according to their frequency within rG4s detected via rG4-seq under K+ conditions. Log2 fold change with respect to the proteomic background is given below. B) Top 20 di- and C) tripeptides encoded by rG4-sequences, sorted according to their frequency within rG4s detected via rG4-seq under K+ conditions.
